# Supplementary material for: Highly luminescent carbazole-functionalized tris(tribromophenyl)methyl radicals with stable circularly polarized photoluminescence
Source: Nat Commun. 2026 May 15;17:4381. doi: 10.1038/s41467-026-73265-z (PMC13179314; doi:10.1038/s41467-026-73265-z)
Supplement: Supplementary file 1 — Supplementary Information [file 41467_2026_73265_MOESM1_ESM.pdf]

Electronic Supplementary Information

Highly Luminescent Carbazole-functionalized Tris(tribromophenyl)methyl Radicals with Stable Circularly Polarized Photoluminescence

Larissa Schöneburg[†,1], Markus Gross[†,1], Philipp Thielert[2,3], Julia Zolg[1,4], Mona E. Arnold[1], Philipp A. Schuster[1], Bernhard Putz[1], Sabine Richert[2,3,]✉ & Alexander J. C. Kuehne[1,4,]✉

1 L. Schöneburg, M. Gross, J. Zolg, Dr. M. E. Arnold, Dr. P. A. Schuster, B. Putz, Prof. Dr. A.J.C. Kuehne
OC III – Institute of Organic and Macromolecular Chemistry
Ulm University
Albert-Einstein-Allee 11, 89081 Ulm, Germany
E-mail: alexander.kuehne@uni-ulm.de

2 P. Thielert, Prof. Dr. S. Richert
Institute of Physical Chemistry II
Ulm University
Albert-Einstein-Allee 11, 89081 Ulm, Germany
Email: sabine.richert@uni-ulm.de

3 P. Thielert, Prof. Dr. S. Richert
Institute of Physical Chemistry
University of Freiburg
Albertstraße 21, 79104 Freiburg, Germany

4 J. Zolg, Prof. Dr. A.J.C. Kuehne
IQST – Center for Integrated Quantum Science and Technology
Ulm University
Albert-Einstein-Allee 11, 89081 Ulm, Germany

[†] these authors contributed equally to this work.

Content

Synthetic Procedures and Spectroscopic Data 3

Synthesis of tris(2,4,6-tribromophenyl)methane – HTTBrM.................... 3

Synthesis of tris(2,4,6-tribromophenyl)methyl radical – TTBrM 4

Synthesis of 9-(4-(bis(2,4,6-tribromophenyl)methyl)-3,5-dibromophenyl)-9*H*-carbazole – HTTBrM-Cz 4

Synthesis of 9-(4-(bis(2,4,6-tribromophenyl)methyl)-3,5-dibromophenyl)-9*H*-carbazole radical - TTBrM-Cz 5

Synthesis of tris(2,6-dibromo-4-(3-methyl-9H-carbazol-9-yl)phenyl)methane – HTTBrM-MeCz 6

Synthesis of tris(2,6-dibromo-4-(3-methyl-9H-carbazol-9-yl)phenyl)methyl radical – TTBrM-MeCz 7

Synthesis of 9-(4-(bis(2,4,6-tribromophenyl)methyl)-3,5-dibromophenyl)-3,6-dimethyl-9*H*-carbazole - HTTBrM-Me_2Cz 7

Synthesis of 9-(4-(bis(2,4,6-tribromophenyl)methyl)-3,5-dibromophenyl)-3,6-dimethyl-9*H*-carbazole radical - TTBrM-Me_2Cz 8

EPR Measurements 8

Time-dependent density functional theory (TD-DFT) calculations.................... 12

Cyclovoltammetry (CV) Measurements 17

Circularly Polarized Luminescence Spectroscopy.................... 17

Thermal stability of the enantiomers 20

Time-correlated single photon counting (TCSPC).................... 21

Photostability.................... 23

Mass spectra.................... 29

References 32

Synthetic Procedures and Spectroscopic Data

Synthesis of tris(2,4,6-tribromophenyl)methane – HTTBrM

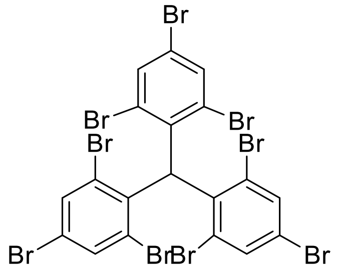

a) In the melt

The Friedel Crafts alkylation is carried out as reported in literature.[1]

A dry high-pressure flask is evacuated and put under an inert atmosphere. 3.00 g 1,3,5-tribromobenzene (9.51 mmol, 5 eq.) and 0.51 g aluminum bromide (1.90 mmol, 1 eq.) are presented, then 0.15 mL anhydrous chloroform are added. The reaction is stirred first at room temperature to avoid too high pressure. Then stirring is continued at 120 °C for three hours. A blue sublimation is observed. During the reaction, the sublimate is melted several times again *via* heat gun to ensure complete implementation of the reactants. Subsequently, the reaction is quenched with cooled 1M HCl and for better solubility, DCM is added. An extraction is carried out with DCM, water and brine before the organic layer is washed with 10% sodium hydrogen carbonate (3 x 25 mL) and dried over magnesium sulphate. Then solvents are removed under reduced pressure and for purification a column chromatography with pure petroleum ether is performed yielding 400 mg of a white solid (0.42 mmol, 22%). For further purification the product is washed with cold petroleum ether and diethyl ether and re-crystallized by cyclohexane.

b) In hexafluorobenzene

A dry high-pressure flask is evacuated and put under an inert atmosphere. 8.25 g 1,3,5-tribromobenzene (26.2 mmol, 7 eq.) are solved in 40 mL hexafluorobenzene. 1.09 g aluminum bromide (4.12 mmol, 1.1 eq.) and 0.3 mL chloroform (3.74 mmol, 1 eq.) are added. The reaction, which changes its color from dark-red to blue, is stirred at 80 °C for 8 d. Subsequently, the reaction is quenched with cooled 1M HCl. An extraction is carried out with DCM, water and brine before the organic layer is dried over magnesium sulphate. Then solvents are removed under reduced pressure and for purification a column chromatography with pure petroleum ether is performed yielding 713 mg of a white solid (0.75 mmol, 20%).

For both, procedure a) and b), the spectroscopic data are in accordance with the literature.[1]

^{1}H NMR (400 MHz, $CDCl_3$): δ [ppm] = 7.75 (d, $^4J_{H,H}$ = 2.1 Hz, 3H), 7.64 (d, $^4J_{H,H}$ = 2.1 Hz, 3H), 6.45 (s, 1H). **HRMS (APCI)**: *m/z* = 953.3111 $[M]^{+\cdot}$ (calculated: 953.3112).

Synthesis of tris(2,4,6-tribromophenyl)methyl radical – TTBrM

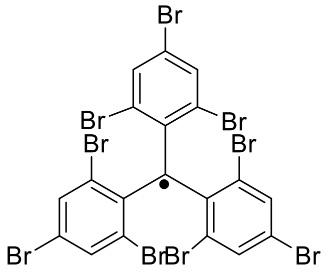

The H-component is converted to the corresponding radical by the established procedure for the synthesis of TTM and PTM.[2]

0.18 g of HTTBrM (0.19 mmol, 1 eq.) are solved in 80 mL anhydrous tetrahydrofuran at room temperature. For deprotonation, 0.42 g potassium tert-butoxide (3.77 mmol, 20 eq.) are added, turning the solution into dark-red and the reaction is stirred overnight. The oxidation to the radical is performed by adding 0.46 g *p* – chloranil (1.89 mmol, 10 eq.). After 3 h, the solvent is removed under reduced pressure and purification *via* column chromatography is carried out with a petroleum ether / DCM 5:1 mixture. 150 mg TTBrM (0.16 mmol, 87%) are obtained as a red solid that shows an orange-red emission under UV irradiation.

Synthesis of 9-(4-(bis(2,4,6-tribromophenyl)methyl)-3,5-dibromophenyl)-9*H*-carbazole – HTTBrM-Cz

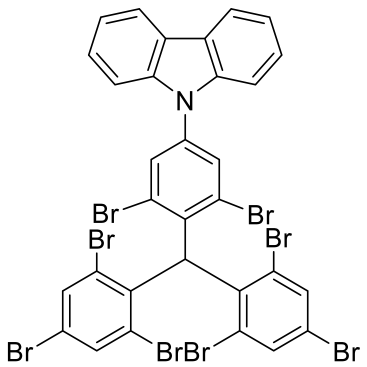

A Buchwald Hartwig cross-coupling reaction is carried out adapted to a reported system containing aza[7]helicene.[3]

109 mg HTTBrM (0.11 mmol, 1 eq.), 28.6 mg carbazole (0.17 mmol, 1.5 eq.), 46.0 mg sodium *tert*-butanolate (0.48 mmol, 4.2 eq.) and 13.3 mg $(^tBu)_3PH{\cdot}BF_4$ (0.05 mmol, 0.4 eq.) are dissolved in 20 mL anhydrous toluene and the mixture is degassed for 30 minutes. Then 20.9 mg $Pd_2(dba)_3$ (0.02 mmol, 0.2 eq.) are added, the reaction mixture is degassed again for 15 min. and is stirred at 50 °C for 3 days. After an extraction over DCM and brine, the organic layer is dried over magnesium sulphate. The solvents are eliminated under reduced pressure. The crude product is purified by column chromatography using a petroleum ether/DCM 5:1 mixture, yielding 65.0 mg HTTBrM-Cz (0.06 mmol, 55%) as a blue solid and 30 mg (0.03 mmol, 23%) of a double functionalized by-product. Further purifications are intended to separate by-products resulting from bromine elimination. A diffusion crystallization in DCM/pentane is performed, the crystals are washed with pentane and dried under vacuum.

HRMS (MALDI) : *m/z* = 1040.4578 $[M]^{+\cdot}$ (calculated: 1040.4588). **^{1}H NMR (600 MHz, CD_2Cl_2)**: δ [ppm] = 8.14 (dt, $^{3}J_{H,H}$ = 7.7, $^{4}J_{H,H}$ = 1.0 Hz, 2H), 7.89 (d, $^{4}J_{H,H}$ = 2.3 Hz, 1H), 7.87 (d, $^{4}J_{H,H}$ = 2.1 Hz, 1H), 7.84 (d, $^{4}J_{H,H}$ = 2.1 Hz, 1H), 7.77 (d, $^{4}J_{H,H}$ = 2.3 Hz, 1H), 7.76 (d, $^{4}J_{H,H}$ = 2.1 Hz, 1H), 7.73 (d, $^{4}J_{H,H}$ = 2.1 Hz, 1H), 7.47 – 7.43 (m, 4H), 7.34-7.31 (m, 2H), 6.67 (s, 1H). **^{13}C NMR (151 MHz, CD_2Cl_2)**: δ [ppm] = 140.49 (s), 138.57 (s), 137.50 (s), 137.39 (s), 137.29 (s), 137.29 (s), 137.26 (s), 137.26 (s), 136.62 (s), 135.75 (s), 135.73 (s), 133.02 (s), 131.32 (s), 129.50 (s), 129.37 (s), 129.36 (s), 128.95 (s), 128.34 (s), 128.20 (s), 128.19 (s), 128.05 (s), 127.36 (s), 126.71 (s), 124.14 (s), 122.15 (s), 121.17 (s), 120.90 (s), 109.92 (s), 65.80 (s), 62.76 (s).

Synthesis of 9-(4-(bis(2,4,6-tribromophenyl)methyl)-3,5-dibromophenyl)-9*H*-carbazole radical - TTBrM-Cz

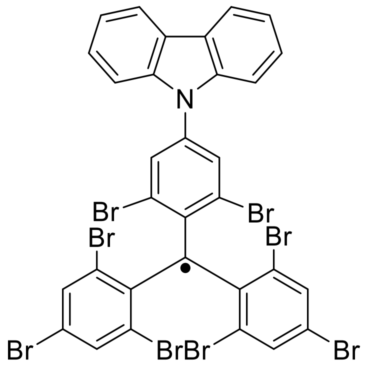

a) The synthesis is carried out in conditions as the known radical mechanism of TTM-type radicals with carbazole.[4]

0.15 g HTTBrM-Cz (0.16 mmol, 1 eq.) and 0.03 g carbazole (0.16 mmol, 1 eq.) are dissolved in 5 mL anhydrous DMF before adding 0.08 g caesium carbonate (0.25 mmol, 1.6 eq.). The reaction is stirred over night at 160 °C in an argon atmosphere.

b) The H-component is converted to the corresponding radical by the established procedure for the synthesis of TTM and PTM.[2]

41.62 mg HTTBrM-Cz (0.04 mmol, 1 eq.) are solved in 20 mL anhydrous THF, 88.64 mg potassium *tert*-butoxide (0.79 mmol, 20 eq.) are added for deprotonation for 24 h. The oxidation to the radical is carried out with 95.89 mg *p*-chloranil (0.39 mmol, 10 eq.) and stirred for 2 h. To filter out excess *p*-chloranil, a rapid column chromatography is carried out with a petroleum ether/DCM 5:1 mixture as eluent. 10.40 mg TTBrM-Cz (0.01 mmol, 25%) are obtained as a green solid which shows an intense red emission under the irradiation of UV-light.

c) Since the classical pathway of the deprotonation-oxidation mechanism using potassium tert-butoxide as base is subject to inefficient conversion in this compound class, potassium hydroxide is used as an alternative together with [18]crown6 as a complexing agent.

5.05 mg HTTBrM-Cz (4.81 µmol, 1 eq.), 5.39 mg mortared potassium hydroxide (95.99 µmol, 20 eq.), and 25.38 mg [18]crown6 (95.99 µmol, 20 eq.) are dissolved in dry THF and stirred for 2 d. Then 11.80 mg *p*-chloranil (47.99 µmol, 10 eq.) are added

and the mixture is stirred for 2 h. To filter out excess *p*-chloranil, a rapid column chromatography is carried out with a petroleum ether/DCM 5:1 mixture as eluent yielding 4.81 mg TTBrM-Cz (4.63 µmol, 96%).

HRMS (MALDI) : *m/z* = 1039.4448 $[M]^{+\cdot}$ (calculated: 1039.4511). **UV-vis:** λ_{max} = 390 and 623 nm. **Fluorescence**: λ_{max} = 646 nm. **Extinction coefficient**: $\varepsilon_{623\,nm}$ = 3630 $l \cdot mol^{-1} cm^{-1}$. **PLQY**: $\Phi_{470\ nm}$ = 72%. **Photostability**: $t_{1/2}$ = 0.96 ± 0.17 s. **Fluorescence lifetime**: τ = 39 ns (argon), 35 ns (O_2). **HPLC**

Synthesis of tris(2,6-dibromo-4-(3-methyl-9H-carbazol-9-yl)phenyl)methane – HTTBrM-MeCz

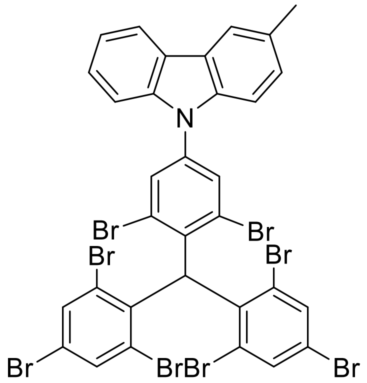

The synthesis is carried out analogously to HTTBrM-Cz.[3]

292 mg HTTBrM (0.31 mmol, 1 eq.), 83.46 mg 3-Methyl-9*H*-carbazole (0.46 mmol, 1.5 eq.), 124 mg sodium *tert*-butanolate (1.29 mmol, 4.2 eq.) and 35.6 mg $(^tBu)_3PH{\cdot}BF_4$ (0.12 mmol, 0.4 eq.) are dissolved in 30 mL anhydrous toluene and the mixture is degassed for 30 minutes. Then 56.2 mg $Pd_2(dba)_3$ (0.06 mmol, 0.2 eq.) are added, the reaction mixture is degassed again for 15 min. and is stirred at 50 °C for 3 days. After an extraction over DCM and brine, the organic layer is dried over magnesium sulphate. The solvents are eliminated under reduced pressure. The crude product is purified by column chromatography using a petroleum ether/DCM 5:1 mixture, yielding 203 mg HTTBrM-MeCz (0.19 mmol, 62%) and multi-functionalized by-products. Further purifications are intended to separate by-products resulting from bromine elimination. A diffusion crystallization in DCM/pentane is performed, the crystals are washed with pentane and dried under vacuum.

HRMS (MALDI) : *m/z* = 1054.506 $[M]^{+\cdot}$ (calculated:). **^{1}H NMR (600 MHz, CD_2Cl_2)**: *δ* [ppm] = 8.10 (dt, $^3J_{H,H}$ = 7.8, $^4J_{H,H}$ = 1.1 Hz, 1H), 7.93 (m, 1H), 7.88 (d, $^4J_{H,H}$ = 2.3 Hz, 1H), 7.86 (d, $^4J_{H,H}$ = 2.1 Hz, 1H), 7.84 (d, $^4J_{H,H}$ = 2.2 Hz, 1H), 7.75 (m, 2H), 7.72 (d, $^4J_{H,H}$ = 2.1 Hz, 1H), 7.47 – 7.40 (m, 2H), 7.35 (d, $^3J_{H,H}$ = 8.3 Hz, 1H), 7.33 – 7.25 (m, 2H), 6.66 (s, 1H), 2.54 (s, 3H). **^{13}C NMR (151 MHz, CD_2Cl_2)**: *δ* [ppm] = 140.76 (s), 138.89 (s), 138.84 (s), 137.58 (s), 137.48 (s), 137.44 (s), 136.39 (s), 135.80 (s), 132.95 (s), 131.24 (s), 130.82 (s), 129.49 (s), 129.44 (s), 129.36 (s), 128.27 (s), 128.21 (s), 128.00 (s), 126.55 (s), 124.25 (s), 124.04 (s), 122.12 (s), 120.99 (s), 120.74 (s), 120.72 (s), 109.98 (s), 109.74 (s), 62.84 (s), 21.48 (s).

Synthesis of tris(2,6-dibromo-4-(3-methyl-9H-carbazol-9-yl)phenyl)methyl radical – TTBrM-MeCz

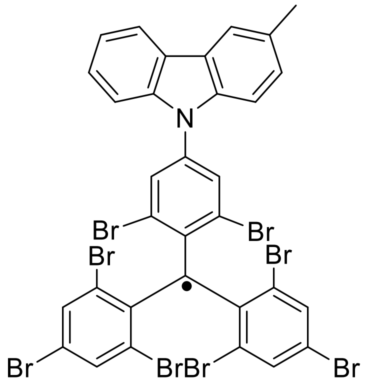

10.00 mg HTTBrM-MeCz (9.48 µmol, 1 eq.), 10.64 mg mortared potassium hydroxide (189 µmol, 20 eq.), and 50.12 mg [18]crown6 (189 µmol, 20 eq.) are dissolved in dry THF and stirred for 2 d. Then 23.32 mg *p*-chloranil (94.8 µmol, 10 eq.) are added and the mixture is stirred for 2 h. To filter out excess *p*-chloranil, a rapid column chromatography is carried out with a petroleum ether/DCM 5:1 mixture as eluent yielding 8.98 mg TTBrM-MeCz (8.53 µmol, 90%).

HRMS (MALDI) : *m/z* = 1053.466 $[M]^{+\cdot}$ (calculated:). **UV-vis:** λ_{max} = 391 and 639 nm. **Fluorescence**: λ_{max} = 679 nm. **Extinction coefficient**: $\varepsilon_{639\,nm}$ = 5571 $l \cdot mol^{-1}cm^{-1}$. **PLQY**: $\Phi_{470\,nm}$ = 70%. **Photostability**: $t_{1/2}$ = 3.22 ± 0.10 s. **Fluorescence lifetime**: τ = 37 ns (argon), 32 ns (O_2). **HPLC**

Synthesis of 9-(4-(bis(2,4,6-tribromophenyl)methyl)-3,5-dibromophenyl)-3,6-dimethyl-9*H*-carbazole - HTTBrM-Me_2Cz

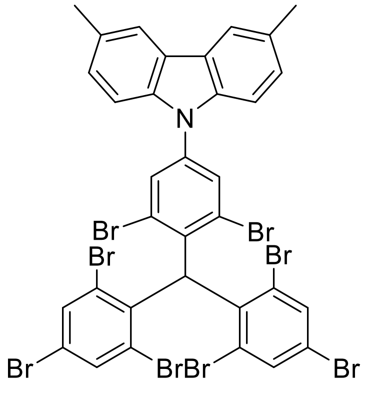

The synthesis is carried out analogously to HTTBrM-Cz and HTTBrM-MeCz.[3]

300 mg HTTBrM (0.31 mmol, 1 eq.), 91.97 mg 3,6-Dimethyl-9*H*-carbazole (0.47 mmol, 1.5 eq.), 127 mg sodium *tert*-butanolate (1.32 mmol, 4.2 eq.) and 36.48 mg $(^tBu)_3PH{\cdot}BF_4$ (0.13 mmol, 0.4 eq.) are dissolved in 30 mL anhydrous toluene and the mixture is degassed for 30 minutes. Then 57.51 mg $Pd_2(dba)_3$ (0.06 mmol, 0.2 eq.) are added, the reaction mixture is degassed again for 15 min. and is stirred at 50 °C for 3 days. After an extraction over DCM and brine, the organic layer is dried over magnesium sulphate. The solvents are eliminated under reduced pressure. The crude product is purified by column chromatography using a petroleum ether/DCM 5:1 mixture, yielding 195 mg HTTBrM-Me_2Cz (0.18 mmol, 59%) and multi-functionalized

by-products. Further purifications are intended to separate by-products resulting from bromine elimination. A diffusion crystallization in DCM/pentane is performed, the crystals are washed with pentane and dried under vacuum.

HRMS (MALDI) : *m/z* = 1068.492 $[M]^{+\cdot}$ (calculated:). **^{1}H NMR (600 MHz, CD_2Cl_2)**: δ [ppm] = 7.89 (s, 2H), 7.87 (d, $^4J_{H,H}$ = 2.3 Hz, 1H), 7.86 (d, $^4J_{H,H}$ = 2.1 Hz, 1H), 7.84 (d, $^4J_{H,H}$ = 2.1 Hz, 1H), 7.76 – 7.73 (m, 2H), 7.72 (d, $^4J_{H,H}$ = 2.2 Hz, 1H), 7.34 (d, $^3J_{H,H}$ = 8.4 Hz, 2H), 7.25 (d, $^3J_{H,H}$ = 8.4 Hz, 2H), 6.65 (s, 1H), 2.53 (s, 6H). **^{13}C NMR (151 MHz, CD_2Cl_2):** δ [ppm] = 139.14 (s), 138.97 (s), 137.58 (s), 137.46 (s), 136.04 (s), 135.80 (s), 135.78 (s), 132.69 (s), 130.99 (s), 130.62 (s), 129.44 (s), 129.35 (s), 128.28 (s), 128.20 (s), 127.82 (s), 124.20 (s), 122.09 (s), 120.65 (s), 109.70 (s), 62.82 (s), 21.47 (s).

Synthesis of 9-(4-(bis(2,4,6-tribromophenyl)methyl)-3,5-dibromophenyl)-3,6-dimethyl-9*H*-carbazole radical - TTBrM-Me_2Cz

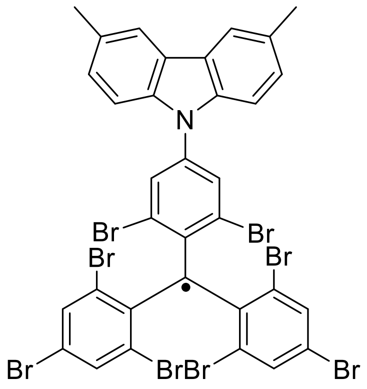

30.0 mg HTTBrM-Me_2Cz (0.03 mmol, 1 eq.), 31.5 mg mortared potassium hydroxide (0.56 mmol, 20 eq.), and 148.3 mg [18]crown6 (0.56 mmol, 20 eq.) are dissolved in dry THF and stirred for 2 d. Then 68.9 mg *p*-chloranil (0.28 mmol, 10 eq.) are added and the mixture is stirred for 2 h. To filter out excess *p*-chloranil, a rapid column chromatography is carried out with a petroleum ether/DCM 5:1 mixture as eluent yielding 29 mg TTBrM-Me_2Cz (0.03 mmol, 99%).

HRMS (MALDI) : *m/z* = 1067.471 $[M]^{+\cdot}$ (calculated:). **UV-vis:** λ_{max} = 391 and 656 nm. **Fluorescence**: λ_{max} = 688 nm. **Extinction coefficient**: $\varepsilon_{656\,\mathrm{nm}}$ = 2997 l · $mol^{-1}cm^{-1}$. **PLQY**: $\Phi_{470\,\mathrm{nm}}$ = 62%. **Photostability**: $t_{1/2}$ = 24.36 $\pm$ 0.95 s. **Fluorescence lifetime**: τ = 33 ns (argon), 27 ns (O_2). **HPLC**

EPR Measurements

Continuous wave (cw) electron paramagnetic resonance (EPR) spectroscopy at the X-band (9.75 GHz) was performed on a Bruker EMXNano benchtop EPR spectrometer. The measurements were carried out in degassed liquid toluene solution (*c* = 0.10 mM) at room temperature in quartz EPR tubes with an outer diameter of 4.95 mm (inner diameter of 4.2 mm). The recorded spectra were frequency-corrected to 9.75 GHz and field-corrected using a carbon fiber standard with g = 2.002644.[5]

Pulse EPR spectroscopy was performed at the Q-band (34 GHz) on a Bruker ELEXSYS E580 EPR spectrometer equipped with a Bruker EN45107D2 resonator. The measurements were carried out in frozen toluene solution (c = 0.10 mM) at 80 K using an Oxford Instruments nitrogen gas-flow cryostat (CF 935) and in quartz EPR tubes with an outer diameter of 1.6 mm (inner diameter of 1 mm).

Electron spin echo (ESE) detected field-swept EPR spectra were recorded using the Hahn-echo sequence, $\pi/2 - \tau - \pi - \tau -$ echo, with a 32 ns π-pulse. The spectra were frequency-corrected to 34 GHz and field-corrected using a carbon fiber standard with g = 2.002644.[5]

The T_1-relaxation data were recorded using a picket-fence sequence composed of 28 saturation pulses (12 ns) and the Hahn-echo detection sequence, using a 32 ns π-pulse and 200 ns inter pulse delay. The data were fit using a biexponential decay function of the form

$$\mathrm{I}(t) = A\exp\left(-\left(\frac{t}{T_1}\right)\right) + \mathrm{A}'\exp\left(-\left(\frac{t}{T_1'}\right)\right) + C.$$

The T_m-relaxation data were recorded using the 2-pulse Hahn-echo sequence, varying the inter pulse delay τ. The data were fit using a stretched monoexponential decay function of the form

$$\mathrm{I}(2t) = A\exp\left(-\left(\frac{2t}{T_\mathrm{m}}\right)^{\beta}\right) + C.$$

While the fitting with a stretched exponential function reproduces the experimental data well, it complicates the direct comparison of the extracted relaxation times T_m, which is why both T_m and β parameters are given.

All samples were left to stabilize for two hours at the measurement temperature of 80 K before the relaxation data were recorded.

Comparative T_m measurements in deuterated frozen toluene solution were carried out analogously, whereby a slightly higher concentration of c = 0.16 mM was used for the reference compound **TTM-Cz**.

To extract the principal g-values of each species, the X- and Q-band spectra were fitted globally using EasySpin (v6.0.5)[6] in MATLAB (vR2023b). The cw X-band spectrum was simulated using the function ‘garlic’ and the pulse-Q-band spectrum was simulated using the function ‘pepper’. The resulting anisotropic g-tensor was averaged to yield the isotropic g-values reported in the main part.

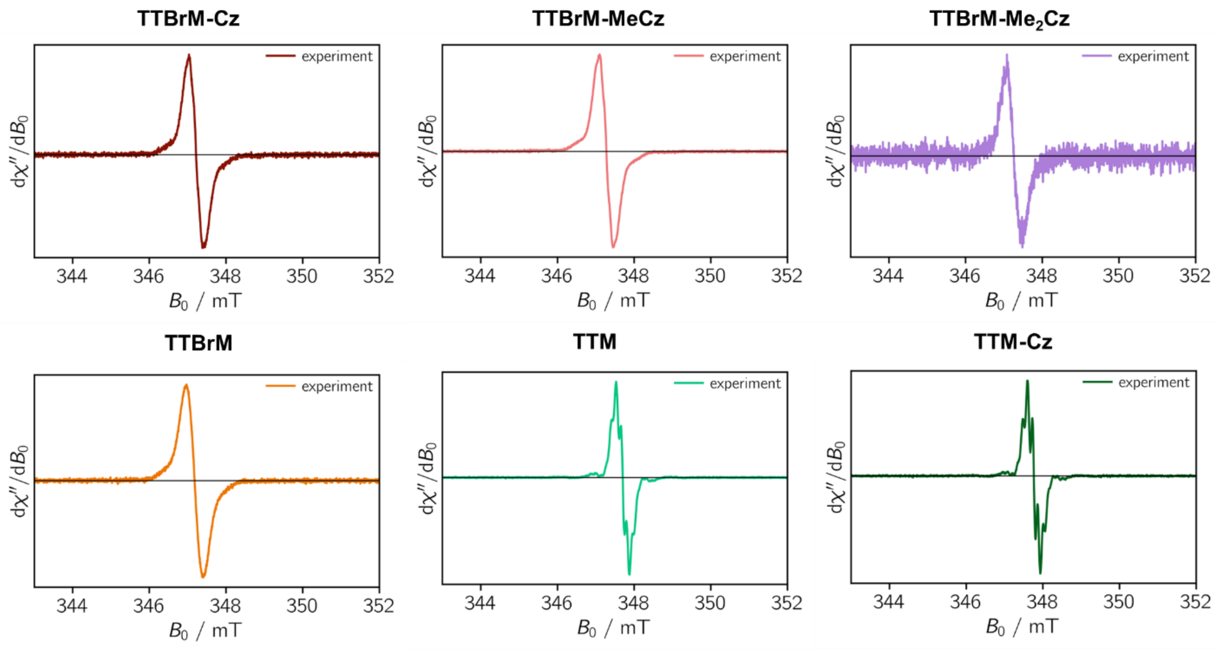

Figure S1: Room temperature cw-EPR measurements in degassed toluene at 26 dB with **TTM**, **TTM-Cz**, and **TTBrM** as reference.

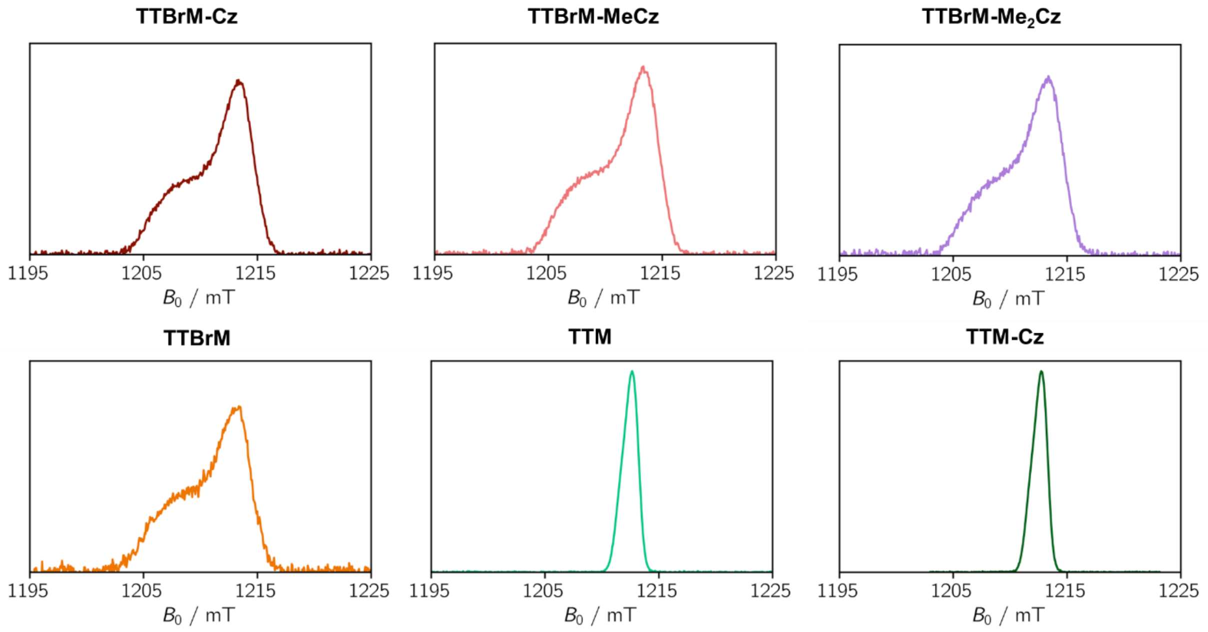

Figure S2: ESE detected field sweep spectra in frozen toluene solution at 80 K with **TTM**, **TTM-Cz**, and **TTBrM** as reference. The anisotropy in the ESE spectra for the TTBrM display the enhanced SOC by the bromine substitution.

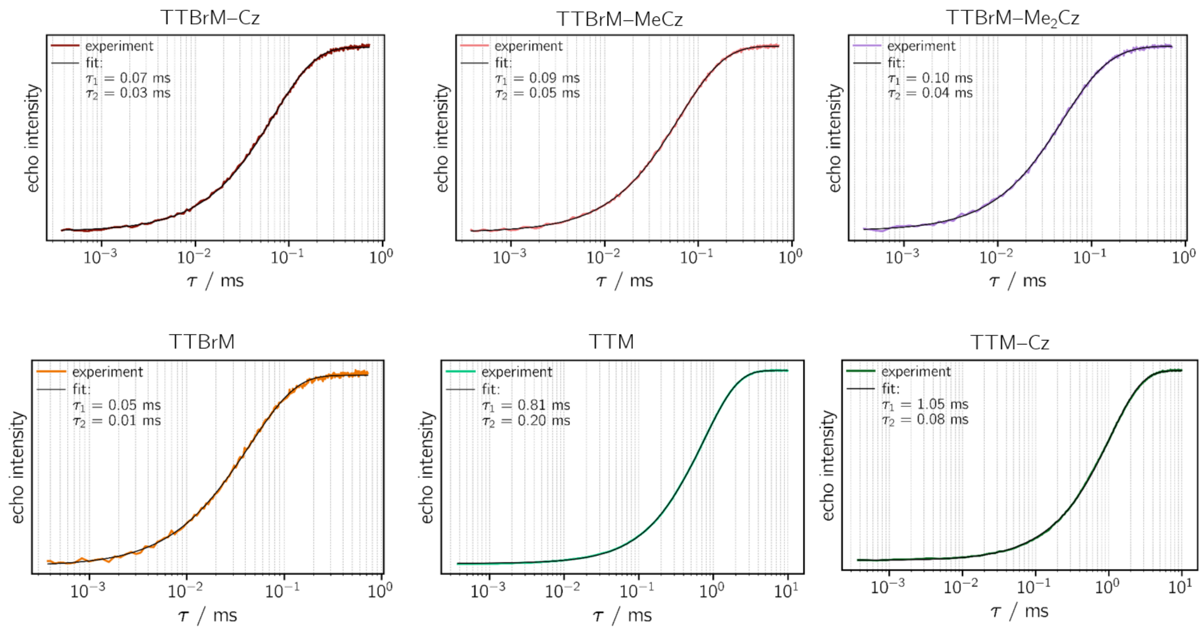

Figure S3: Picket fence T_1 relaxation data in frozen toluene solution (c = 0.1 mM, 80 K) with a biexponential decay fitted to the experimental data to determine T_1, shown in logarithmic scale.

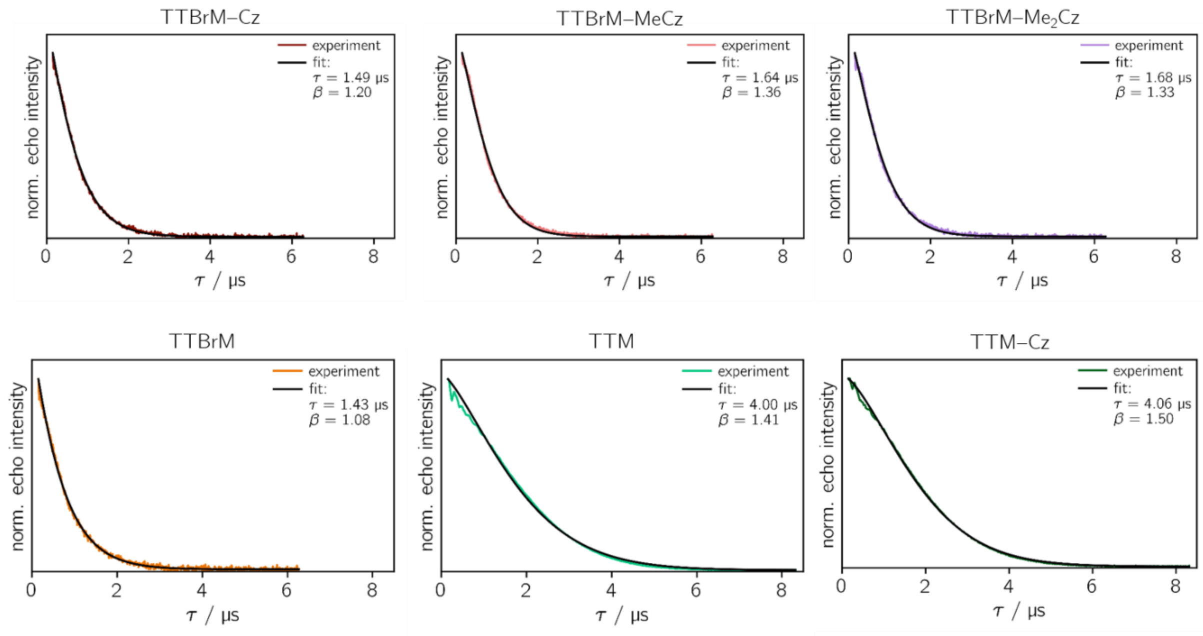

Figure S4: Spin coherence decay data in frozen toluene solution (c = 0.1 mM, 80 K) with a stretched exponential decay fitted to the experimental data to determine T_m.

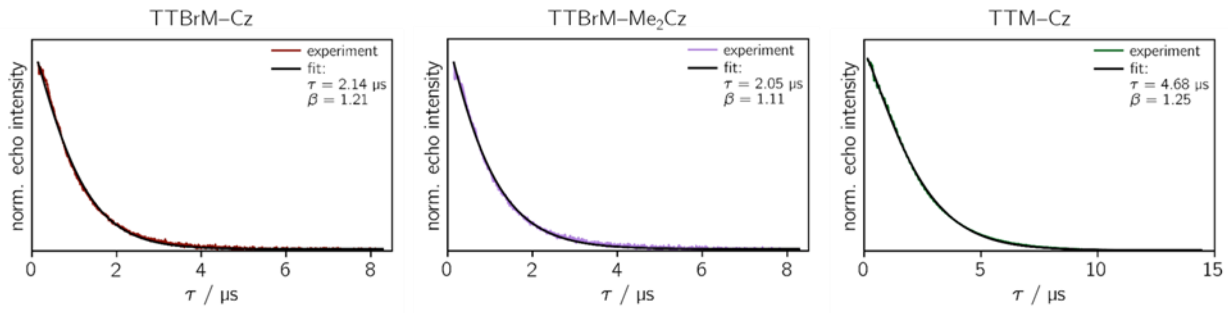

Figure S5: Spin coherence decay data in deuterated frozen toluene solution (c = 0.1 mM, 0.16 mM for **TTM-Cz**, 80 K) with a stretched exponential decay fitted to the experimental data to determine T_m.

Time-dependent density functional theory (TD-DFT) calculations

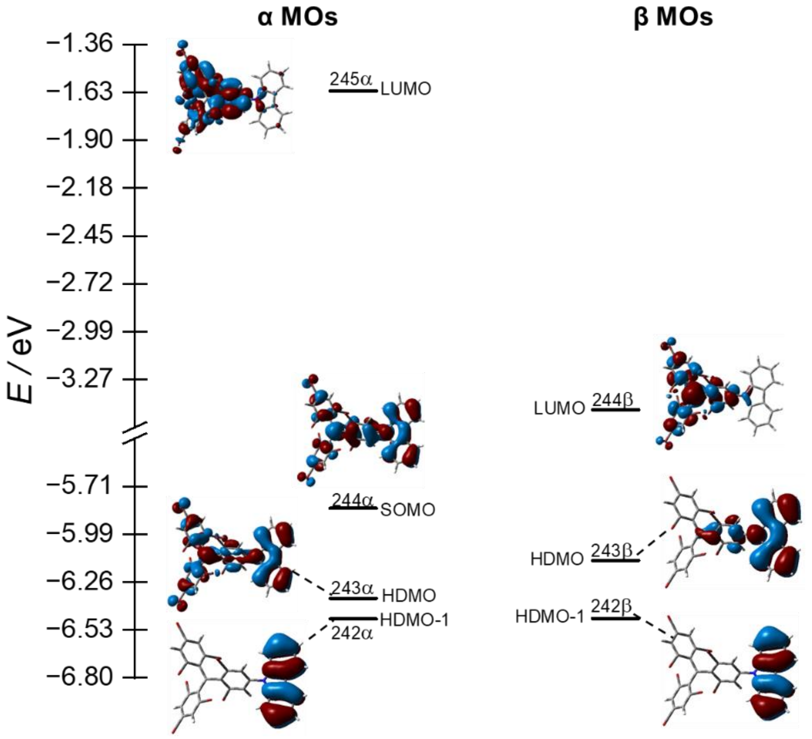

Figure S6: Ground state orbital distributions and corresponding energy levels of **TTBrM-Cz**.

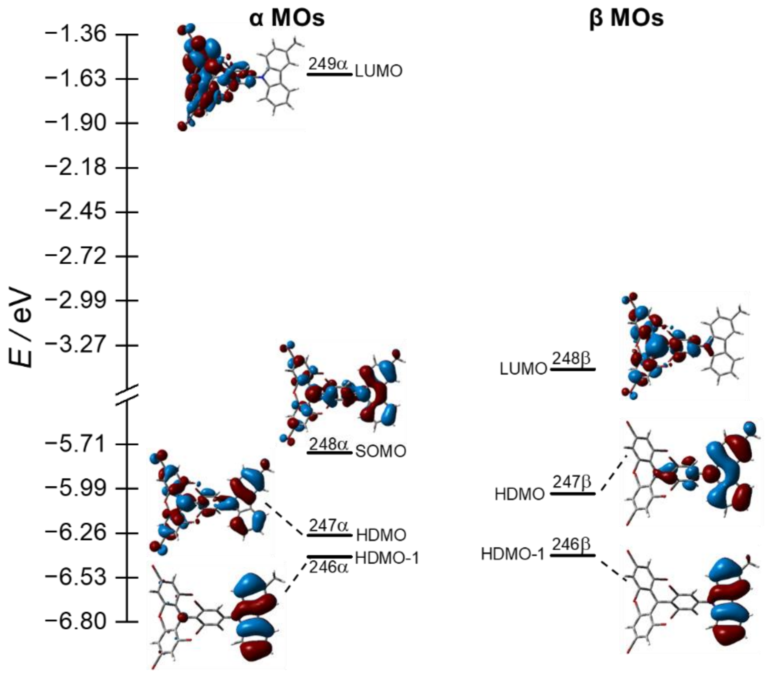

Figure S7: Ground state orbital distributions and corresponding energy levels of **TTBrM-MeCz**.

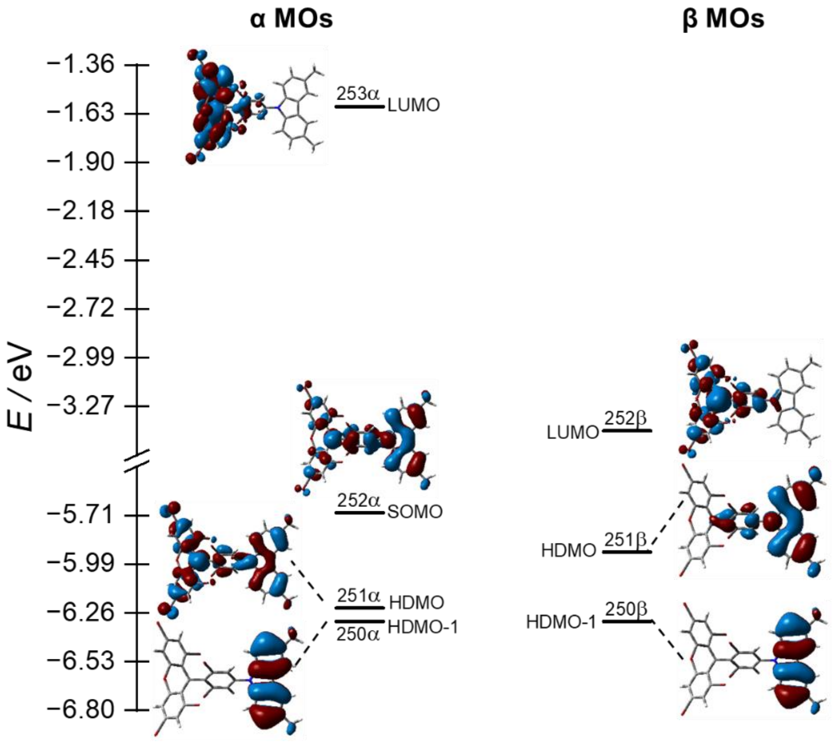

Figure S8: Ground state orbital distributions and corresponding energy levels of **TTBrM-Me$_2$Cz**.

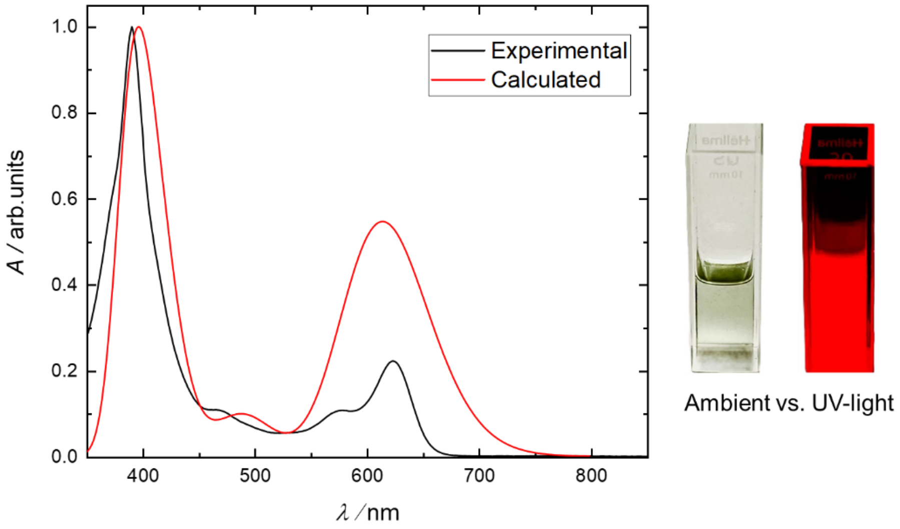

Figure S9: Comparison of the experimental UV-Vis data of **TTBrM-Cz** with the computed spectrum obtained by TD-DFT (left) and the color of the solution at ambient light vs. under UV irradiation in cyclohexane (right).

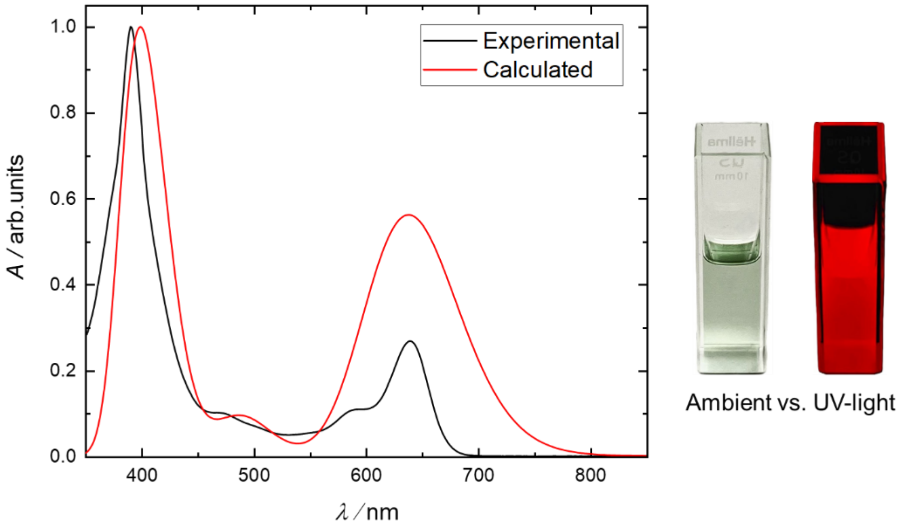

Figure S10: Comparison of the experimental UV-Vis data of **TTBrM-MeCz** with the computed spectrum obtained by TD-DFT (left) and the color of the solution at ambient light vs. under UV irradiation in cyclohexane (right).

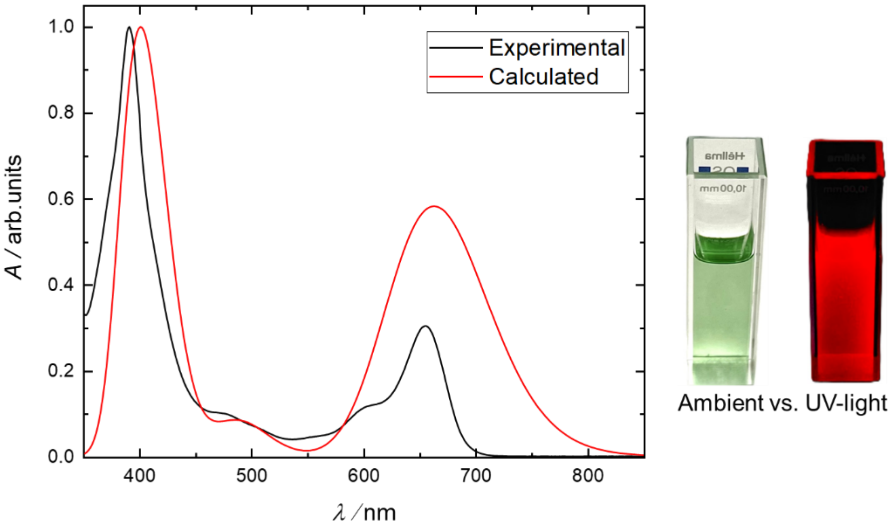

Figure S11: Comparison of the experimental UV-Vis data of **TTBrM-Me$_2$Cz** with the computed spectrum obtained by TD-DFT (left) and the color of the solution at ambient light vs. under UV irradiation in cyclohexane (right).

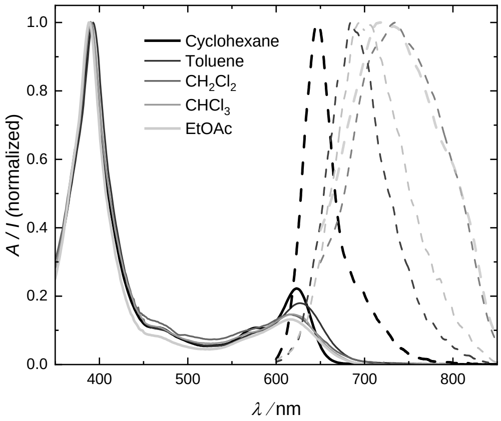

Figure S12: Solvent-polarity-dependent absorption and emission spectra (excitation wavelength λ_{ex} = 400 nm) of **TTBrM-Cz**. The spectra have been normalized to enable better comparability of the emission maxima and spectral shapes.

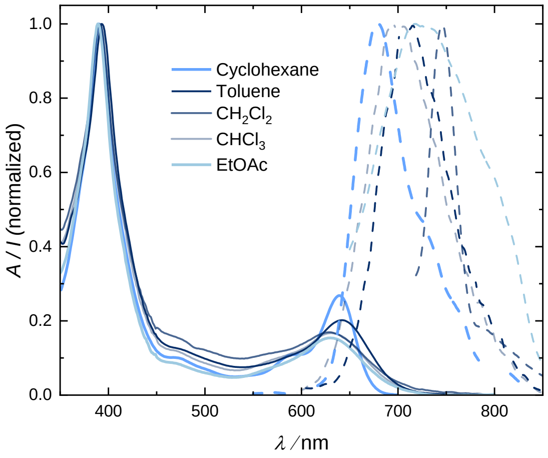

Figure S13: Solvent-polarity-dependent absorption and emission spectra (excitation wavelength λ_{ex} = 400 nm) of **TTBrM-MeCz**. The spectra have been normalized to enable better comparability of the emission maxima and spectral shapes.

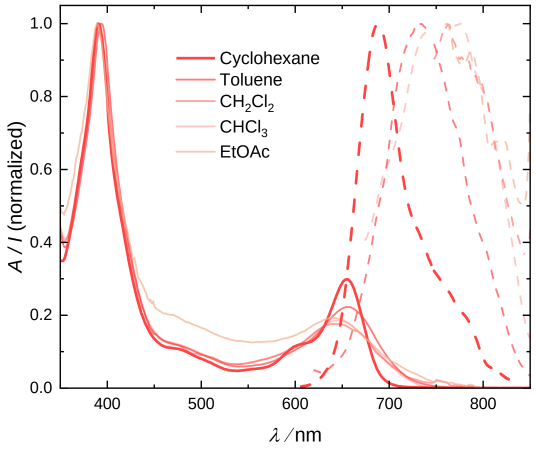

Figure S14: Solvent-polarity-dependent absorption and emission spectra (excitation wavelength λ_{ex} = 400 nm) of **TTBrM-Me$_2$Cz**. The spectra have been normalized to enable better comparability of the emission maxima and spectral shapes.

Cyclovoltammetry (CV) Measurements

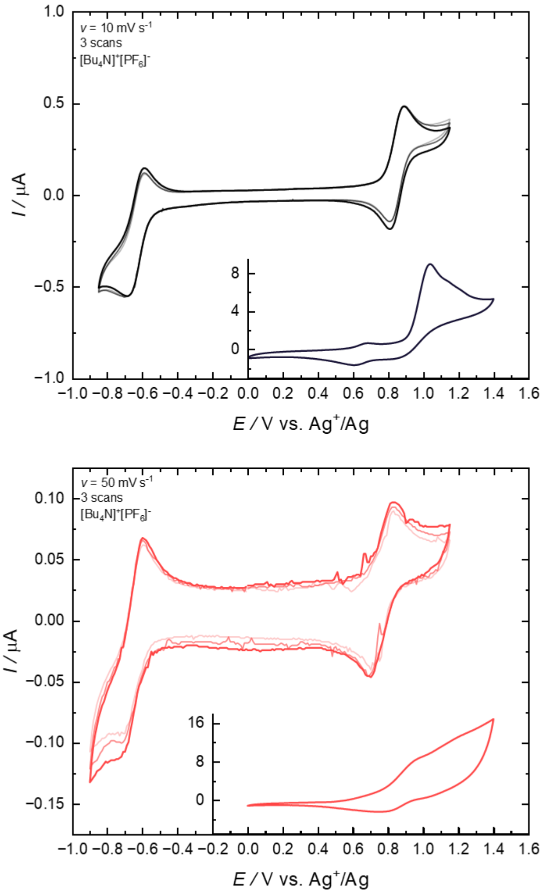

Figure S15: CV measurements in methylene chloride of **TTBrM-Cz** (black), **TTBrM-MeCz** (blue), and **TTBrM-Me$_2$Cz** (red) in a three-electrode-setup with an Ag/Ag$^+$ reference electrode and $[Bu_4N]^+[PF_6]^-$ (0.1 M) as electrolyte. The insets display the cyclic voltammograms of the corresponding donors.

Circularly Polarized Luminescence Spectroscopy

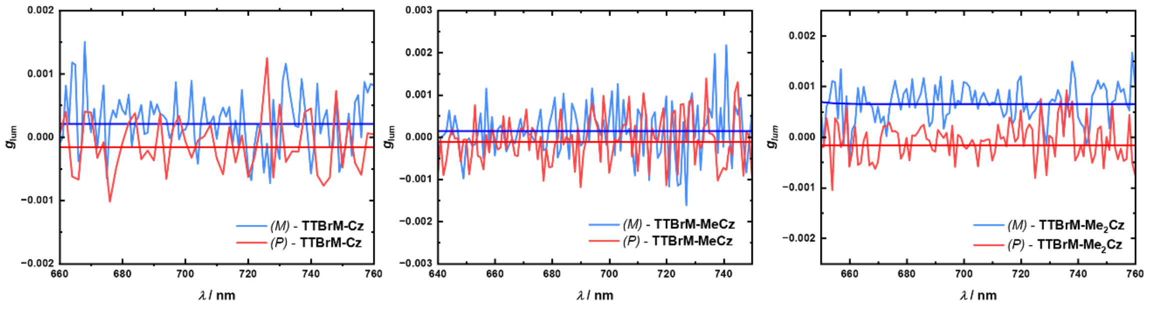

Figure S16: Circularly Polarized Luminescence Spectra of **TTBrM-Cz, TTBrM-MeCz,** and **TTBrM-Me$_2$Cz** all in toluene (293 K, c~10^{-5} M). Final dissymmetry factors (g_{lum}) are obtained by calculating the mean in the area of emission.

Table S1 Experimental dissymmetry factors (g_{lum}) of *(M)* and *(P)* light emitting radicals.

**TTBrM-Cz**	g_{lum} / 10^{-4}	**TTBrM-MeCz**	g_{lum} / 10^{-4}	**TTBrM-Me2Cz**	g_{lum} / 10^{-4}
(M)	2.5	*(M)*	1.5	*(M)*	3.5
(P)	5.3	*(P)*	5.1	*(P)*	6.5

Table S2 Summary of the calculated dissymmetry factors for absorption (g_{abs}) and emission (g_{lum}) of the (*P*)-enantiomer radicals. $|\mu_e|$ is the electric transition dipole moment, $|\mu_m|$ is the magnetic transition dipole moment, θ is the angle between the electric and magnetic transition dipole moment vectors.

	Absorption (ground state geometry)				*Photoluminescence (excited state geometry)*			
(*P*)	$\|\mu_e\|$ / 10^{-20} esu·cm	$\|\mu_m\|$ / 10^{-20} erg G^{-1}	θ / °	g_{abs} 10^{-5}	$\|\mu_e\|$ / 10^{-20} esu·cm	$\|\mu_m\|$ / 10^{-20} erg G^{-1}	θ / °	g_{lum} 10^{-4}
**TTBrM-Cz**	382	0.0034	2.59	3.01	350	0.0147	3.14	1.68
**TTBrM-MeCz**	402	0.0189	1.57	0.07	202	0.0269	2.79	4.97
**TTBrM-Me2Cz**	424	0.0025	0.99	1.31	207	0.0252	3.14	4.87

Chromatography

Table S3 Overview of retention times of the different enantiomers.

**Compound**	Retention time Fraction *(M)*	Retention time Fraction *(P)*
**TTBrM-Cz**	23.2	20.2
**TTBrM-MeCz**	11.2	7.5
**TTBrM-Me2Cz**	21.3	19.2

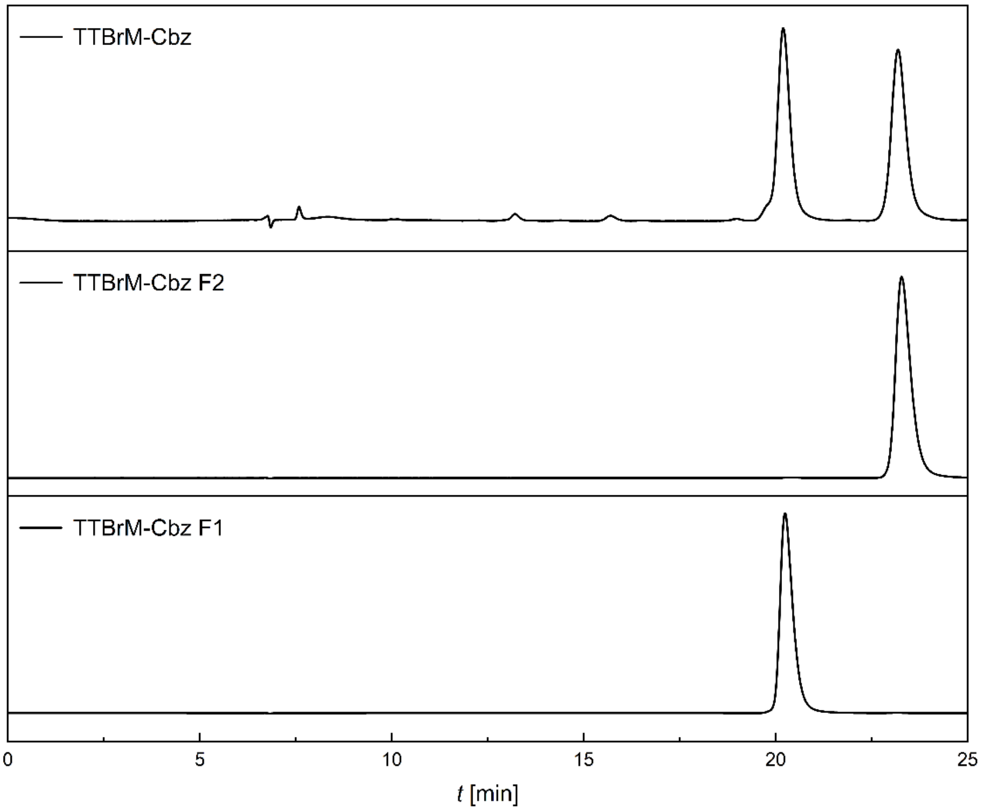

Figure S17: (CSP)HPLC chromatogram of **TTBrM-Cz** using a chiral stationary phase column. A Daicel Chiralpak® IB N-3 3 µm (250 x 4.6mm) was used with an injection volume of 50 µL of a 1mg/mL solution in n-hexane / chloroform 80:20 and a flow rate of 0.5mL/min. A mixture of 80:20 n-hexane / chloroform was used as eluent.

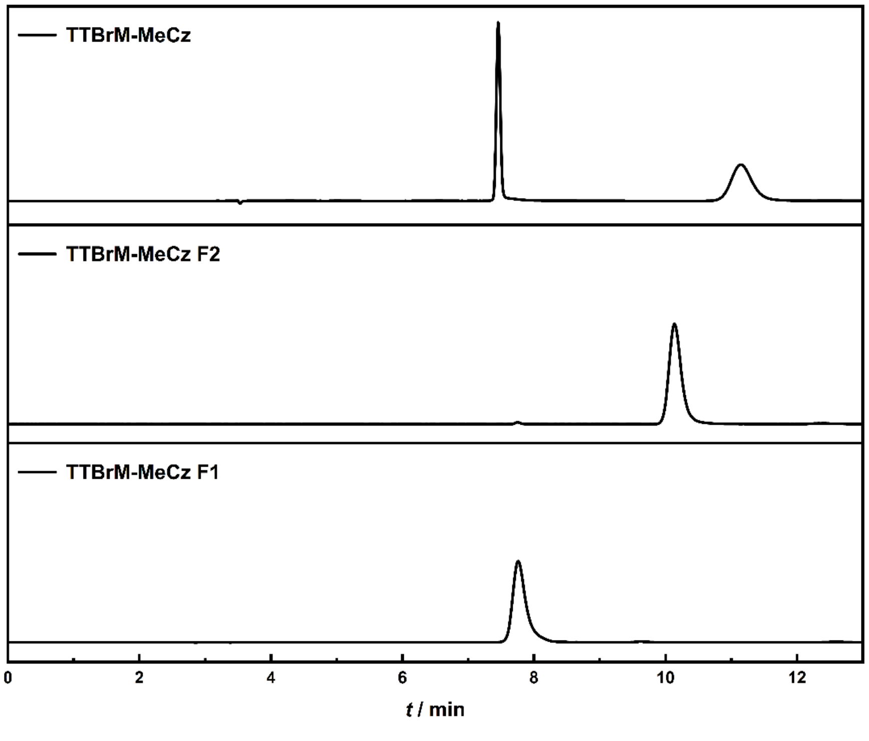

Figure S18: (CSP)HPLC chromatogram of **TTBrM-MeCz** using a chiral stationary phase column. A Daicel Chiralpak® IB N-3 3 µm (250 x 4.6mm) was used with an injection volume of 50 µL of a 1mg/mL solution in n-hexane / chloroform / isopropanol 80:19.5:0.5 and a flow rate of 1mL/min. A mixture of 80:19.5:0.5 n-hexane / chloroform / isopropanol was used as eluent.

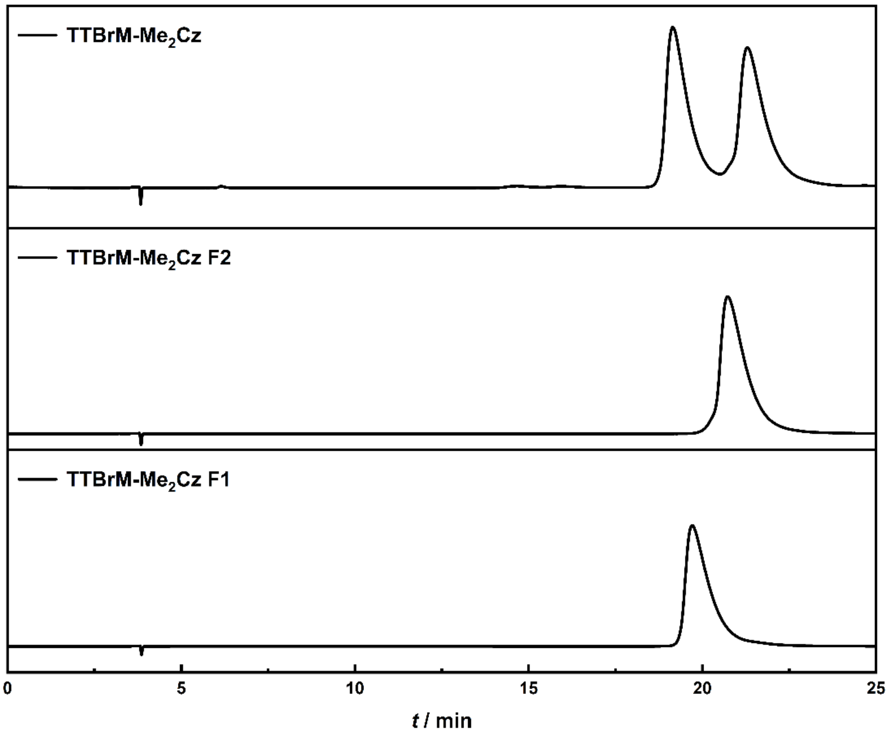

Figure S19: (CSP)HPLC chromatogram of **TTBrM-Me$_2$Cz** using a chiral stationary phase column. A Daicel Chiralpak® IB N-3 3 µm (250 x 4.6mm) was used with an injection volume of 50 µL of a 1mg/mL solution in n-hexane / chloroform 90:10 and a flow rate of 1mL/min. A mixture of 90:10 n-hexane / chloroform was used as eluent.

Thermal stability of the enantiomers

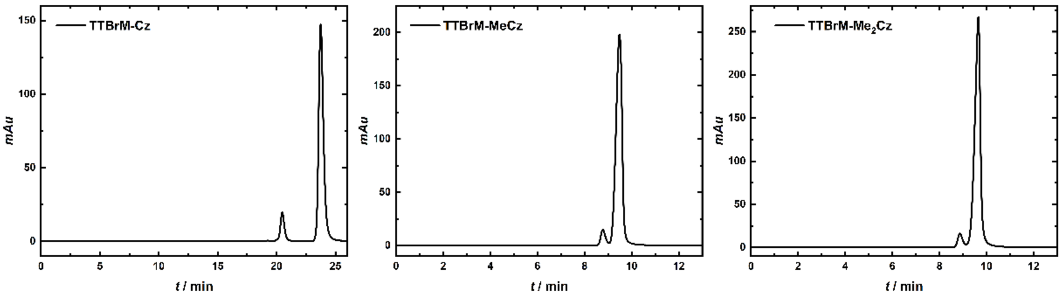

Figure S20: Chromatograms of **TTBrM-Cz**, **TTBrM-MeCz** and **TTBrM-Me$_2$Cz** after being heated at a temperature of 80°C in cyclohexane for 4 hours and being reinjected into the HPLC. **TTBrM-MeCz** and **TTBrM-Me$_2$Cz** chromatograms were run on a Phenomenex Lux i-Amylose-3, 5 µm (250 x 4.6mm). **TTBrM-Cz** was run on a Daicel Chiralpak® IB N-3 3 µm (240 x 4.6mm).

Time-correlated single photon counting (TCSPC)

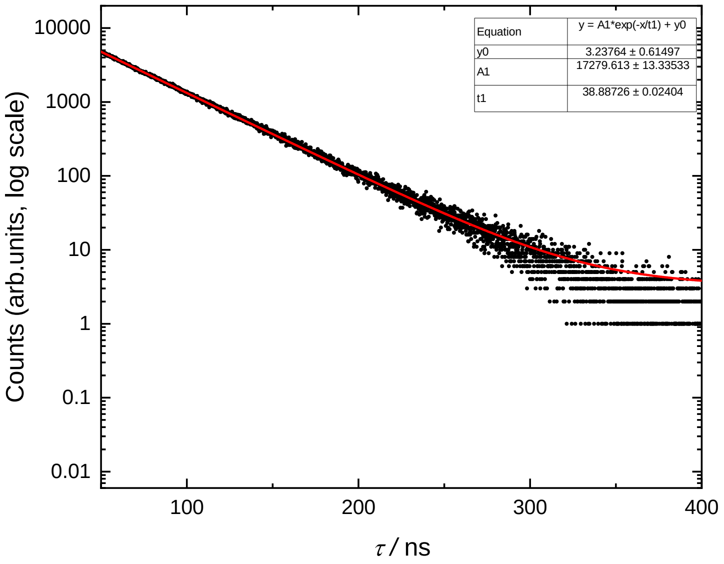

Figure S21: TCSPC measurement of a degassed **TTBrM-Cz** solution to obtain the PL lifetime.

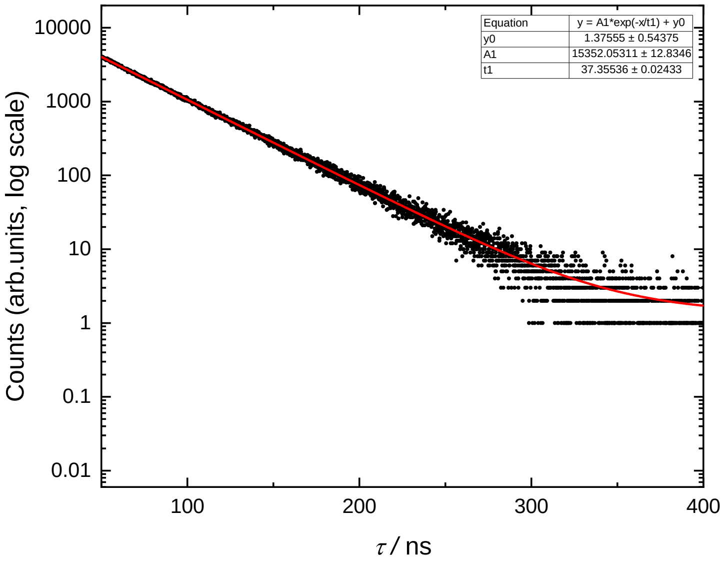

Figure S22: TCSPC measurement of a degassed **TTBrM-MeCz** solution to obtain the PL lifetime.

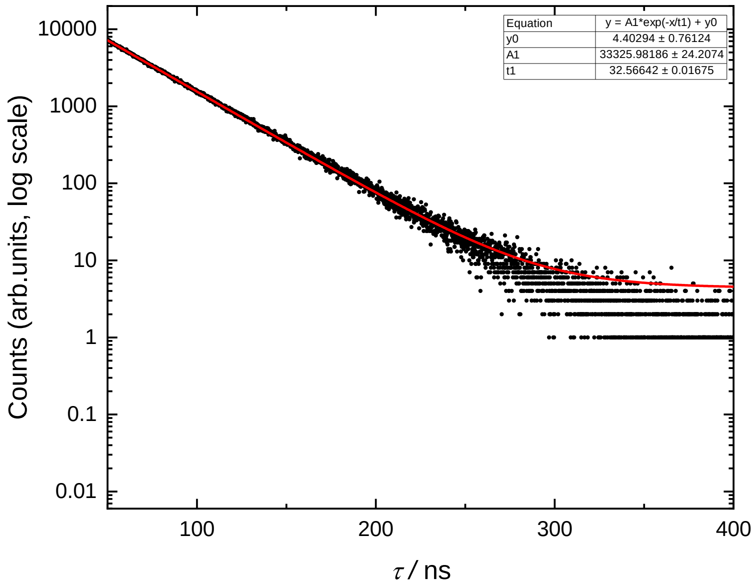

Figure S23: TCSPC measurement of a degassed **TTBrM-Me$_2$Cz** solution to obtain the PL lifetime.

Photostability

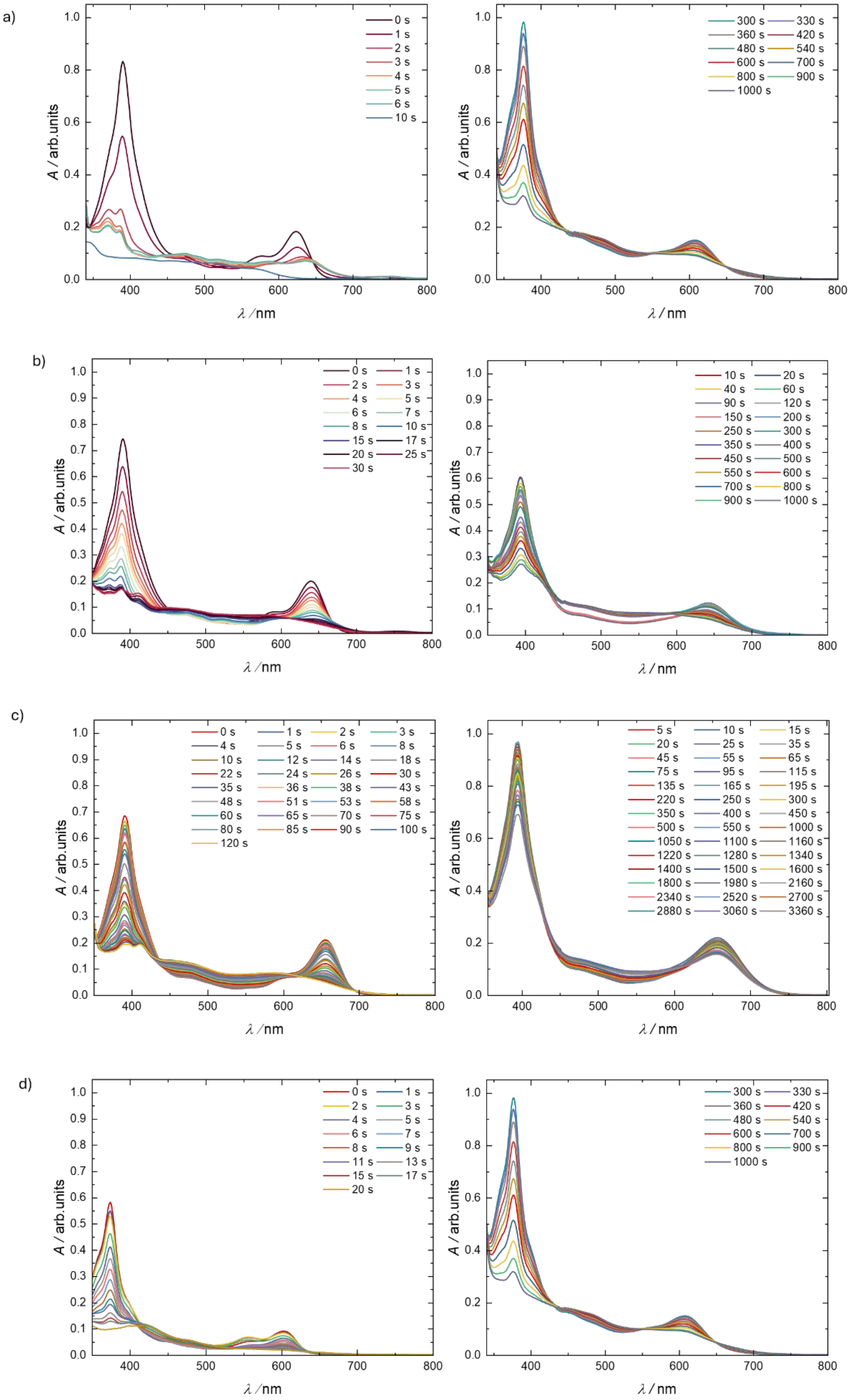

Figure S24: UV-Vis absorption spectra of a degassed solution in cyclohexane (left) and toluene (right) recorded after different UV-irradiation times of a) **TTBrM-Cz**, b) **TTBrM-MeCz**, c) **TTBrM-Me$_2$Cz**, and d) **TTM-Cz**.

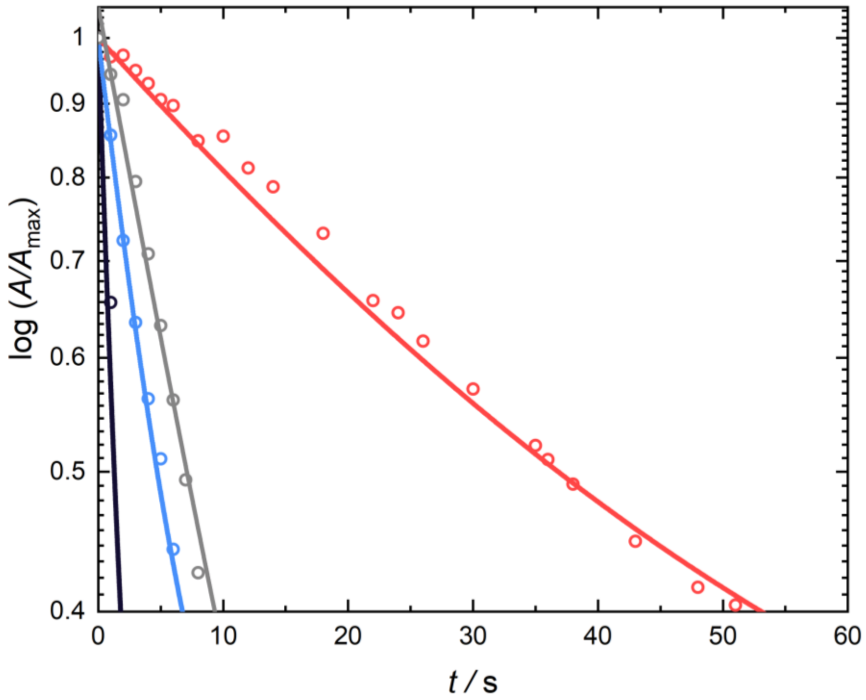

Figure S25: Time-dependent decrease of a selected absorption band, plotted together with an exponential decay fit in a logarithmic scale to extract the photochemical half-life time in cyclohexane of **TTBrM-Cz** (black), **TTBrM-MeCz** (blue), **TTBrM-Me$_2$Cz** (red), and **TTM-Cz** (grey).

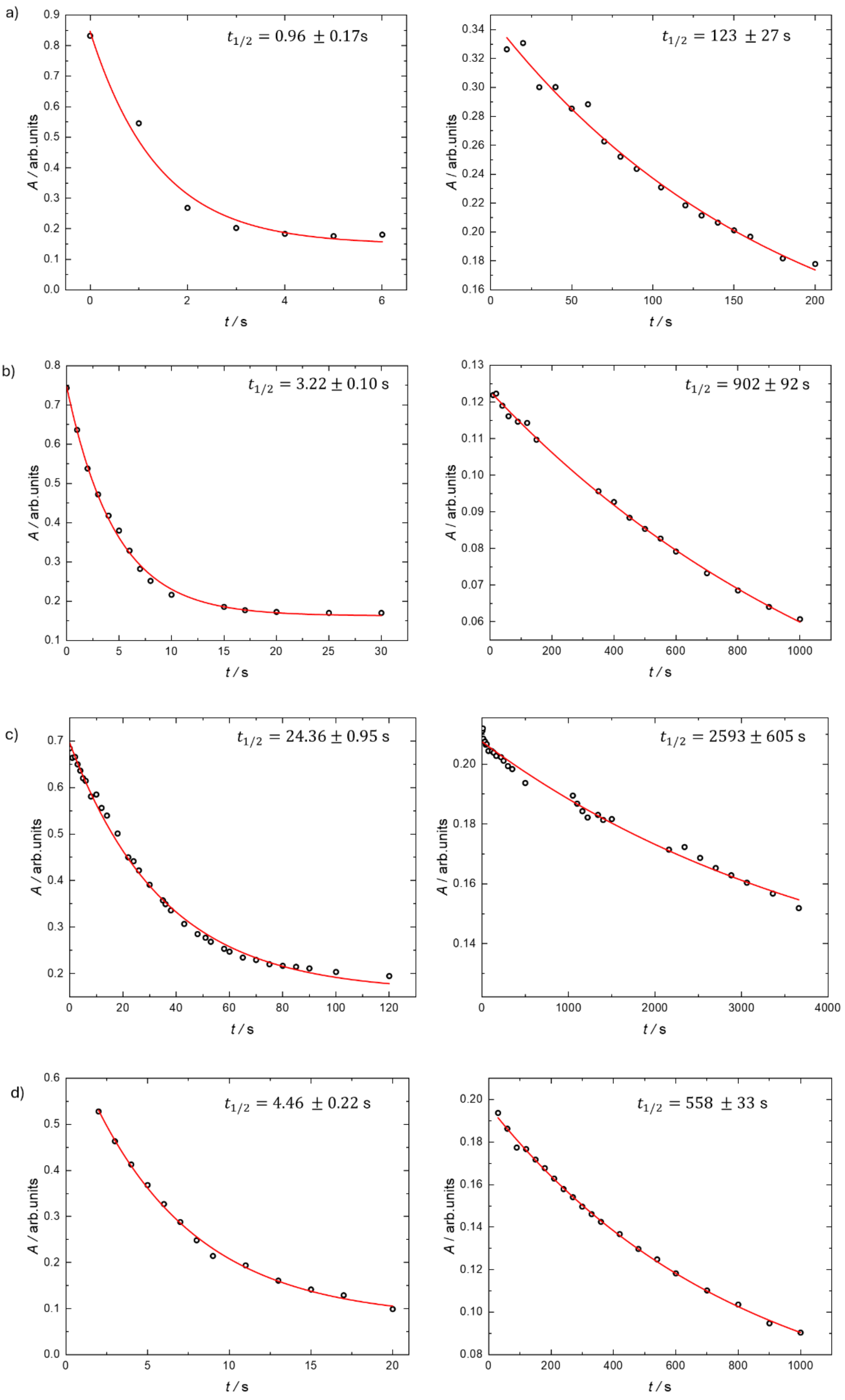

Figure S26: Time-dependent decrease of a selected absorption band, plotted together with an exponential decay fit to extract the photochemical half-life time (inset) in degassed cyclohexane (left) and toluene (right) of a) **TTBrM-Cz**, b) **TTBrM-MeCz**, c) **TTBrM-Me$_2$Cz**, and d) **TTM-Cz**.

NMR spectra

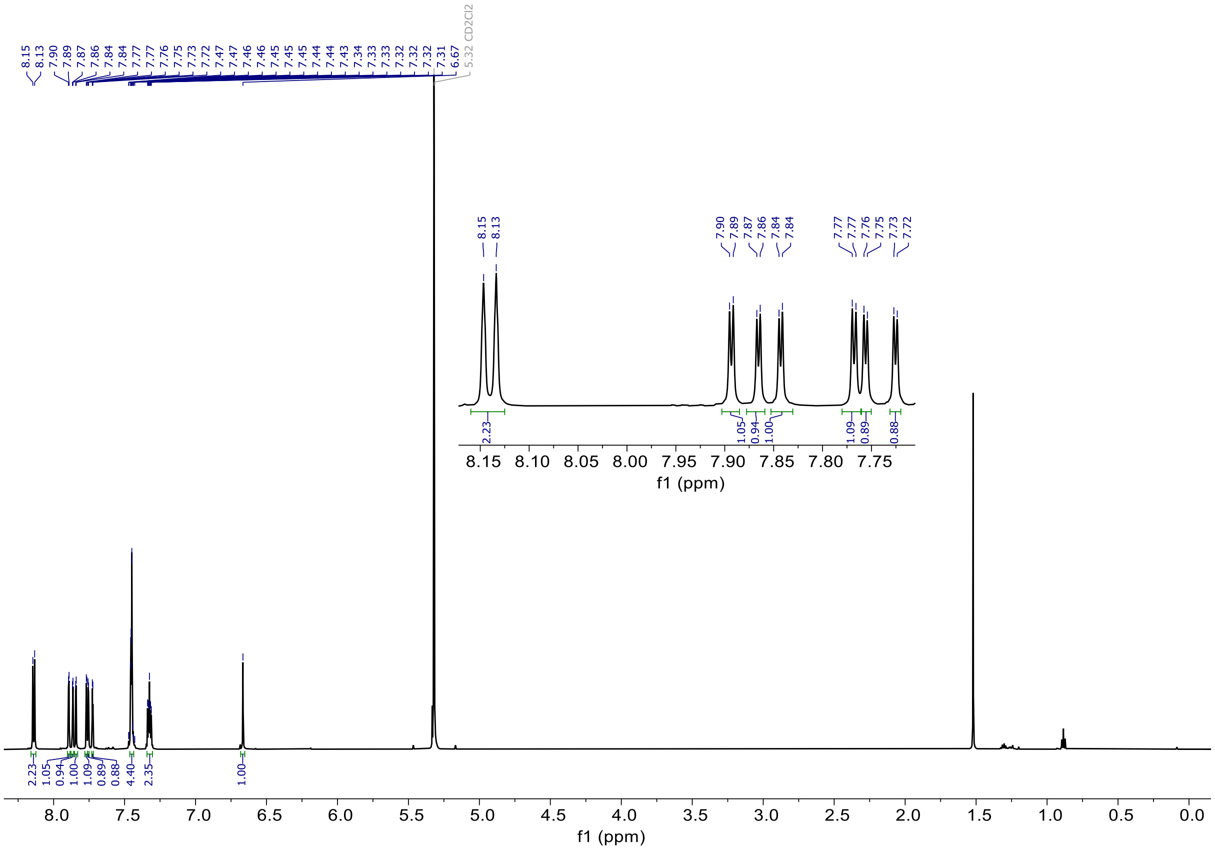

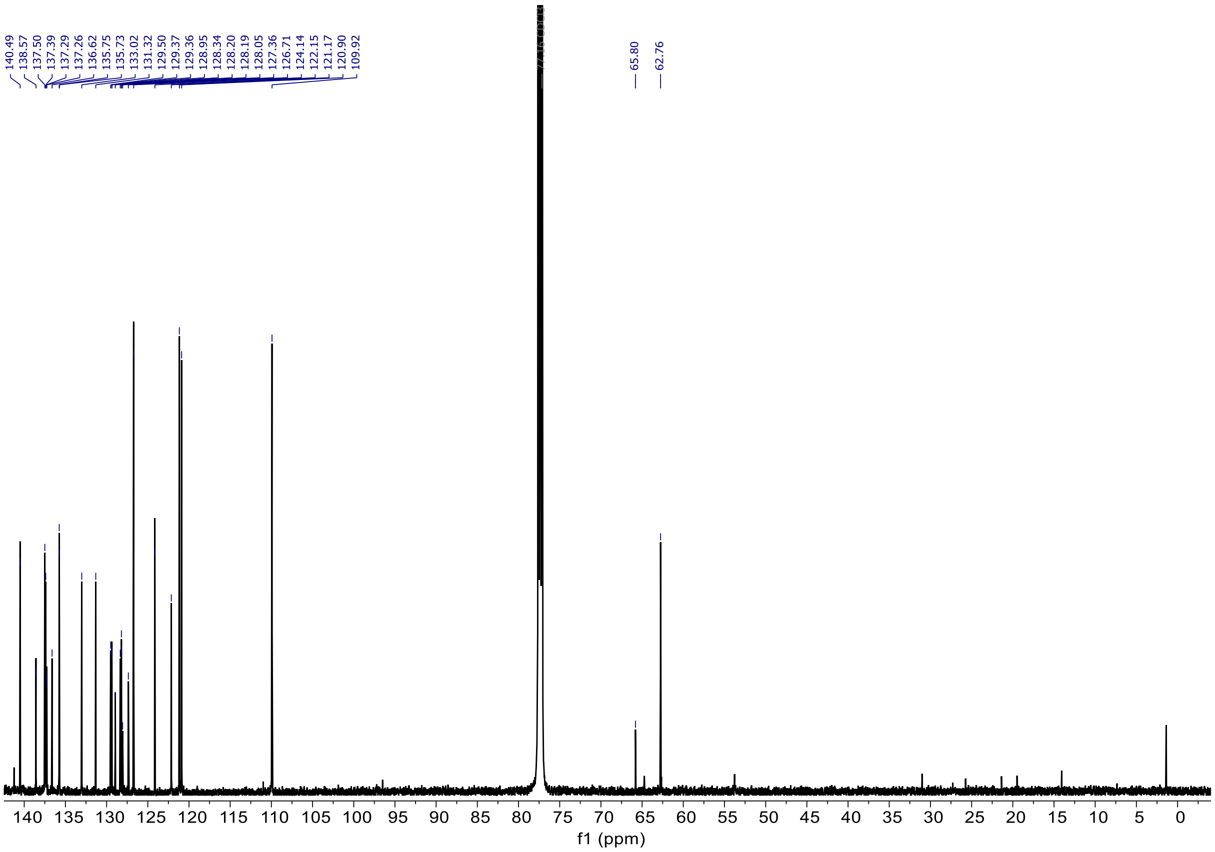

Figure S27: ^{1}H (top) (600 MHz) and ^{13}C-NMR (600 MHz) (bottom) spectra of **HTTBrM-Cz** in CD_2Cl_2.

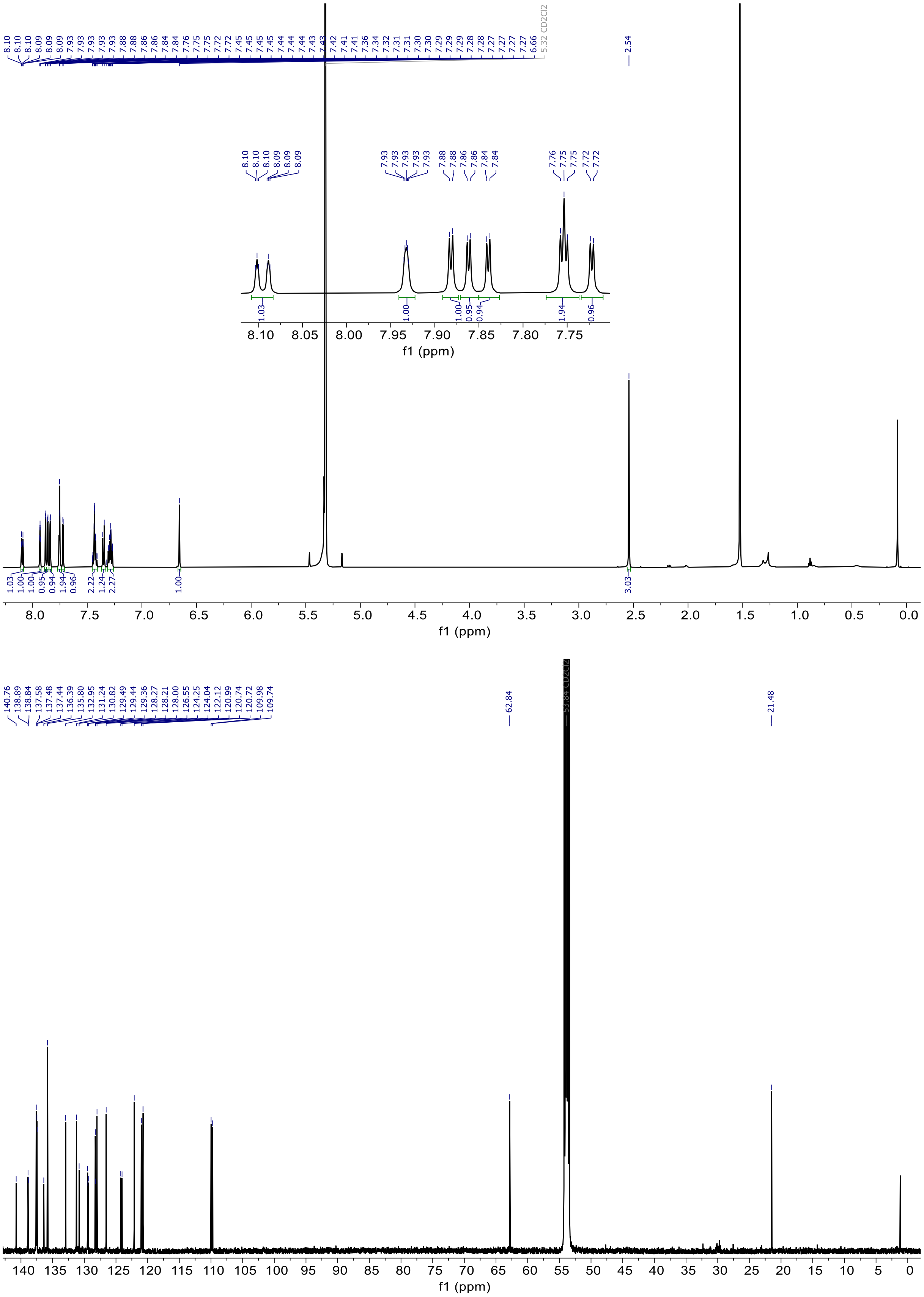

Figure S28: ^{1}H (top) (600 MHz) and ^{13}C-NMR (600 MHz) (bottom) spectra of **HTTBrM-MeCz** in CD_2Cl_2.

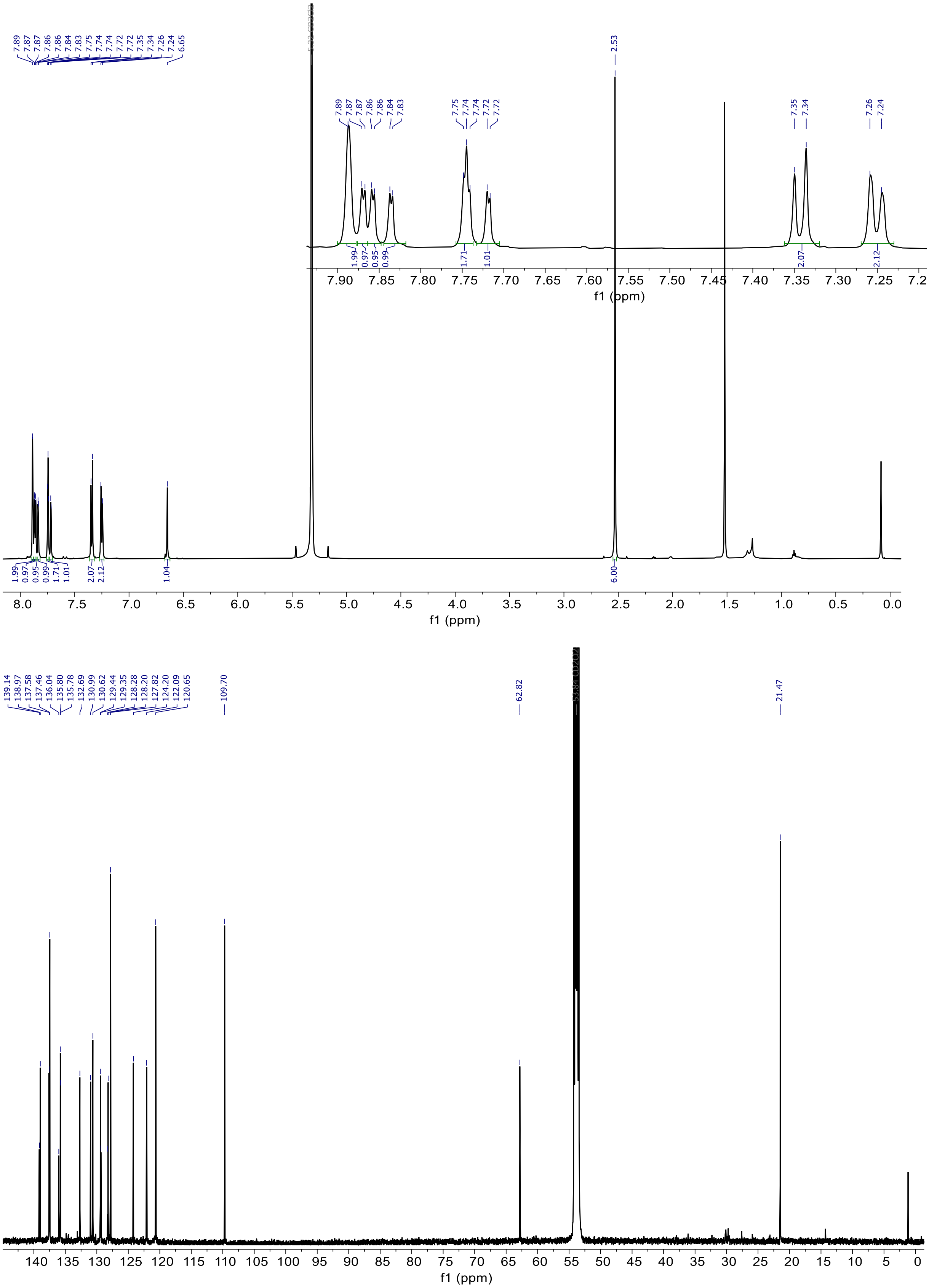

Figure S29: ^{1}H (top) (600 MHz) and ^{13}C-NMR (bottom) (600 MHz) spectra of **HTTBrM-Me$_2$Cz** in CD_2Cl_2.

Mass spectra

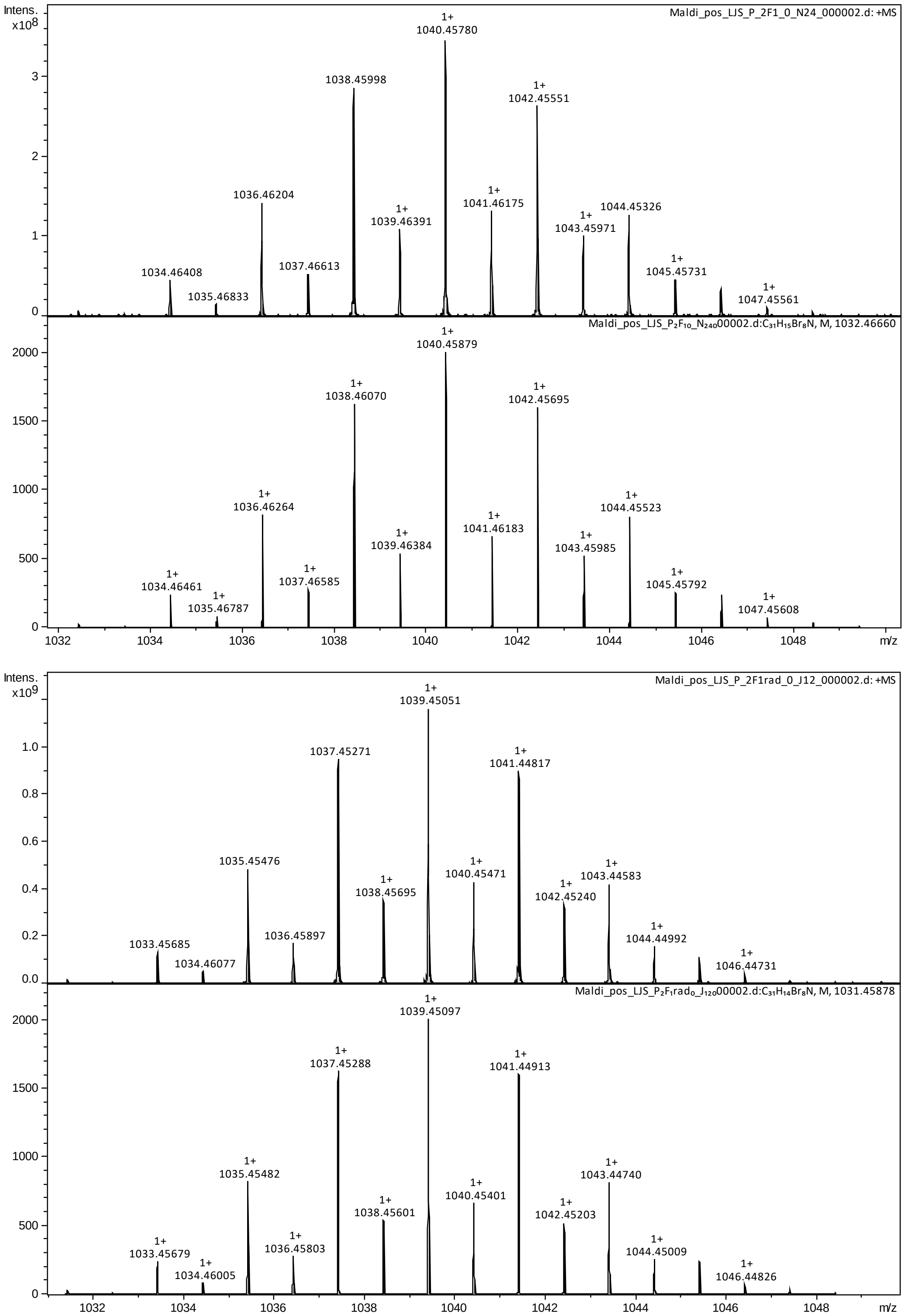

Figure S30: HRMS (Maldi, positive mode) and calculated mass spectrum of **HTTBrM-Cz** (top) and **TTBrM-Cz** (bottom).

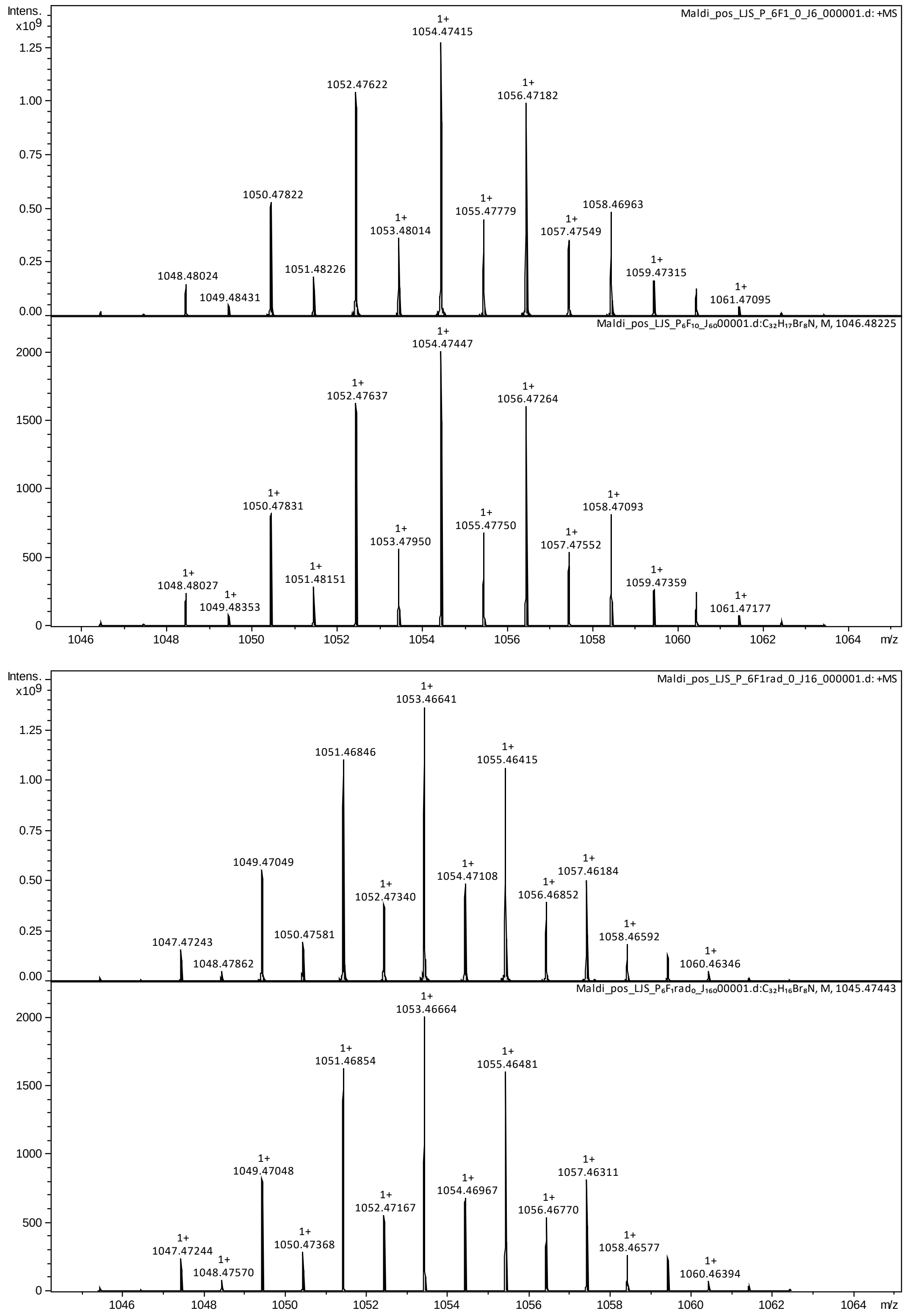

Figure S31: HRMS (Maldi, positive mode) and calculated mass spectrum of **HTTBrM-MeCz** (top) and **TTBrM-MeCz** (bottom).

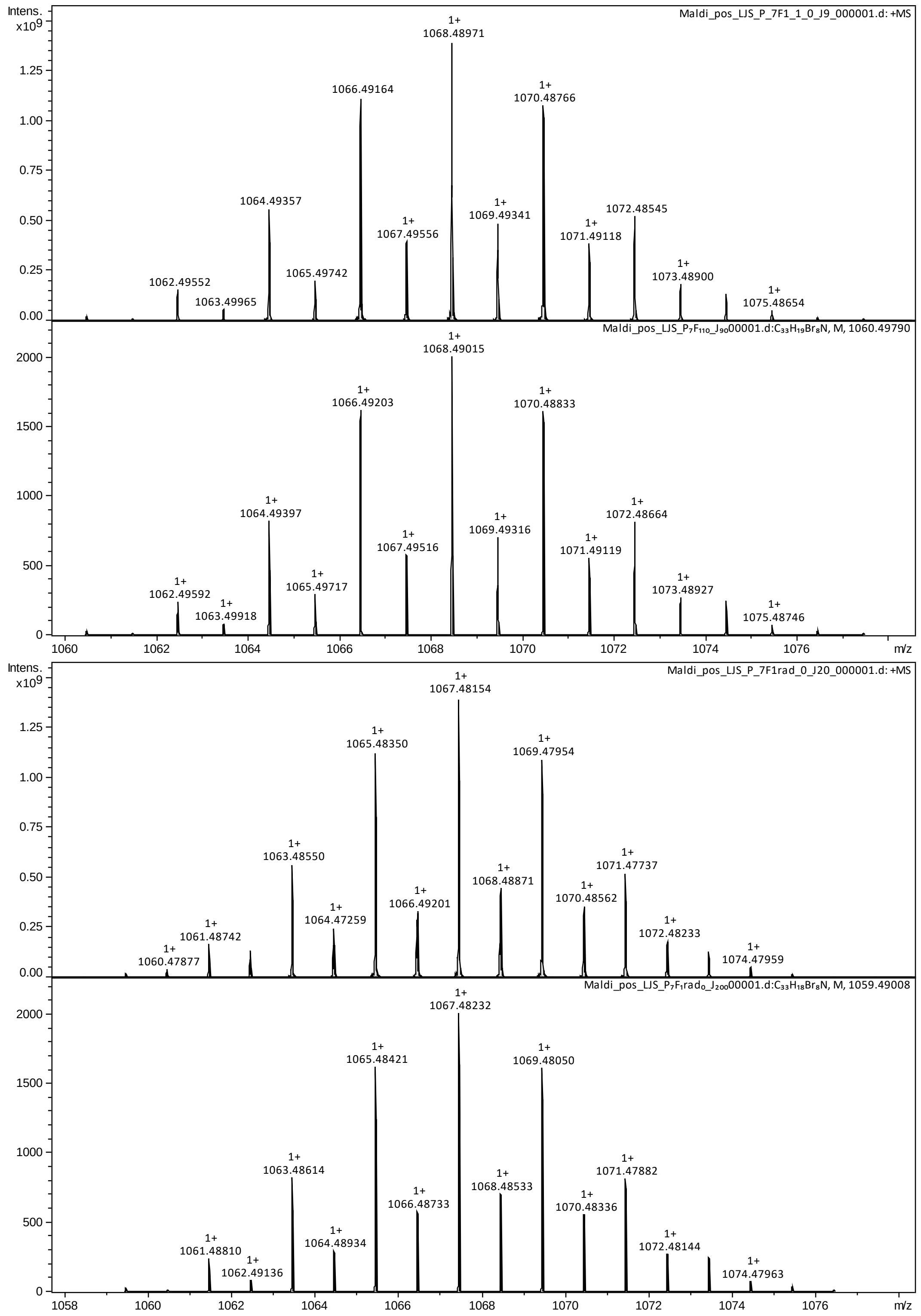

Figure S32: HRMS (Maldi, positive mode) and calculated mass spectrum of **HTTBrM-Me$_2$Cz** (top) and **TTBrM-Me$_2$Cz** (bottom).

References

[1] P. Mayorga-Burrezo, V. G. Jiménez, D. Blasi, T. Parella, I. Ratera, A. G. Campaña, J. Veciana, "An Enantiopure Propeller-Like Trityl-Brominated Radical: Bringing Together a High Racemization Barrier and an Efficient Circularly Polarized Luminescent Magnetic Emitter" *Chem. - A Eur. J.* **2020**, *26*, 3776–3781.

[2] X. Ai, E. W. Evans, S. Dong, A. J. Gillett, H. Guo, Y. Chen, T. J. H. Hele, R. H. Friend, F. Li, "Efficient radical-based light-emitting diodes with doublet emission" *Nature* **2018**, *563*, 536–540.

[3] M. Gross, F. Zhang, M. E. Arnold, P. Ravat, A. J. C. Kuehne, "Aza[7]helicene Functionalized Triphenylmethyl Radicals with Circularly Polarized Doublet Emission" *Adv. Opt. Mater.* **2024**, *12*, 2301707.

[4] D. Velasco, S. Castellanos, M. López, F. López-Calahorra, E. Brillas, L. Juliá, "Red organic light-emitting radical adducts of carbazole and tris(2,4,6-trichlorotriphenyl)methyl radical that exhibit high thermal stability and electrochemical amphotericity" *J. Org. Chem.* **2007**, *72*, 7523–7532.

[5] K. Herb, R. Tschaggelar, G. Denninger, G. Jeschke, "Double resonance calibration of g factor standards: Carbon fibers as a high precision standard" *J. Magn. Reson.* **2018**, *289*, 100–106.

[6] S. Stoll, A. Schweiger, "EasySpin, a comprehensive software package for spectral simulation and analysis in EPR" *J. Magn. Reson.* **2006**, *178*, 42–55.

[7] M. J. Frisch, G. W. Trucks, H. B. Schlegel, G. E. Scuseria, M. a. Robb, J. R. Cheeseman, G. Scalmani, V. Barone, G. a. Petersson, H. Nakatsuji, X. Li, M. Caricato, a. V. Marenich, J. Bloino, B. G. Janesko, R. Gomperts, B. Mennucci, H. P. Hratchian, J. V. Ortiz, a. F. Izmaylov, J. L. Sonnenberg, Williams, F. Ding, F. Lipparini, F. Egidi, J. Goings, B. Peng, A. Petrone, T. Henderson, D. Ranasinghe, V. G. Zakrzewski, J. Gao, N. Rega, G. Zheng, W. Liang, M. Hada, M. Ehara, K. Toyota, R. Fukuda, J. Hasegawa, M. Ishida, T. Nakajima, Y. Honda, O. Kitao, H. Nakai, T. Vreven, K. Throssell, J. a. Montgomery Jr., J. E. Peralta, F. Ogliaro, M. J. Bearpark, J. J. Heyd, E. N. Brothers, K. N. Kudin, V. N. Staroverov, T. a. Keith, R. Kobayashi, J. Normand, K. Raghavachari, a. P. Rendell, J. C. Burant, S. S. Iyengar, J. Tomasi, M. Cossi, J. M. Millam, M. Klene, C. Adamo, R. Cammi, J. W. Ochterski, R. L. Martin, K. Morokuma, O. Farkas, J. B. Foresman, D. J. Fox, **2016**, Gaussian, Inc.
